# Review of the Southeast Asian millipede genus *Antheromorpha* Jeekel, 1968 (Diplopoda, Polydesmida, Paradoxosomatidae)

**DOI:** 10.3897/zookeys.571.7566

**Published:** 2016-03-07

**Authors:** Natdanai Likhitrakarn, Sergei I. Golovatch, Somsak Panha

**Affiliations:** 1 Division of Plant Protection, Faculty of Agricultural Production, Maejo University, Chiang Mai 50290, Thailand; 2 Institute for Problems of Ecology and Evolution, Russian Academy of Sciences, Leninsky pr. 33, Moscow 119071, Russia; 3 Animal Systematics Research Unit, Department of Biology, Faculty of Science, Chulalongkorn University, Bangkok 10330, Thailand

**Keywords:** Review, Paradoxosomatidae, taxonomy, new synonymy, key, distribution, map

## Abstract

The genus *Antheromorpha* is redefined and shown to comprise 11 valid species: *Antheromorpha miranda* (Pocock, 1895), *Antheromorpha bistriata* (Pocock, 1895), *Antheromorpha comotti* (Pocock, 1895), *Antheromorpha festiva* (Brölemann, 1896), *Antheromorpha harpaga* (Attems, 1937), *Antheromorpha mediovirgata* (Carl, 1941), *Antheromorpha minlana* (Pocock, 1895), *Antheromorpha pardalis* (Pocock, 1895), *Antheromorpha paviei* (Brölemann, 1896), **comb. n.**, *Antheromorpha rosea* Golovatch, 2013 and *Antheromorpha uncinata* (Attems, 1931). Three new synonymies are proposed: *Antheromorpha bivittata* (Pocock, 1895) and *Antheromorpha melanopleuris* (Pocock, 1895) are synonymized under *Antheromorpha miranda* (Pocock, 1895), and *Antheromorpha orophila* (Carl, 1941) under *Antheromorpha comotti* (Pocock, 1895). Detailed descriptions and illustrations of fresh material from Thailand and Malaysia are given, especially regarding colour patterns which appear to be crucial for accurate species identifications. Two *Antheromorpha* species proposed by Attems are redescribed, based on type material. The genus is rediagnosed and a key and a distribution map are also provided. At least in Thailand, adult *Antheromorpha rosea* have been found to occur every year only for one or two weeks in September or October, disappearing thereafter.

## Introduction

The Southeast Asian millipede genus *Antheromorpha* Jeekel, 1968 is currently known to comprise 13 medium-sized to very large species showing moderately developed to very prominent paraterga and, above all, unlike the other, numerous genera of the basically Oriental tribe Orthomorphini Brölemann, 1916 to which it belongs, a very deeply bifid gonopod tip (Jeekel 1968, Golovatch 2013a). This genus is assumed to be particularly similar to the largely sympatric genus *Orthomorpha* Bollman, 1893, the species of which, like *Antheromorpha*, normally have large bodies and prominent paraterga, coupled with usually bright colour patterns. The main difference between these two genera lies in *Orthomorpha* spp. showing only a poorly differentiated gonopod tip, usually feebly bi- or trifid (Likhitrakarn et al. 2011). *Antheromorpha* species have hitherto been recorded in Myanmar (9), Thailand (2), China (1) and Vietnam (1).

Because of the remarkably bright colour patterns and large bodies, unidentified *Antheromorpha* species have repeatedly been reported throughout Thailand (http://siamensis.org/webboard/topic/35582#comment-34142; http://thailandwildlife.photoshelter.com/gallery-image/Other-Arthropods/G0000OdCpTbz8ENY/I0000PMJm_Wnsl8E; http://www.projectnoah.org/spottings/10657453; https://www.flickr.com/photos/lennyworthington/sets/72157628909951579/). Moreover, one of the species shows swarming behaviour (http://www.manager.co.th/Local/ViewNews.aspx?NewsID=9490000084506).

The only attempt at reviewing *Antheromorpha* and outlining its diagnosis (Jeekel 1980) considers the following species arranged in six species groups:

1. *Antheromorpha bistriata* (Pocock, 1895), *Antheromorpha bivittata* (Pocock, 1895), *Antheromorpha festiva* (Brölemann, 1896), *Antheromorpha mediovirgata* (Carl, 1941), *Antheromorpha melanopleuris* (Pocock, 1895), *Antheromorpha miranda* (Pocock, 1895).
2. *Antheromorpha comotti* (Pocock, 1895), *Antheromorpha orophila* (Carl, 1941).
3. *Antheromorpha uncinata* (Attems, 1931).
4. *Antheromorpha harpaga* (Attems, 1937).
5. *Antheromorpha minlana* (Pocock, 1895).
6. *Antheromorpha pardalis* (Pocock, 1895).

The only subsequent addition to the list seems to be *Antheromorpha rosea* Golovatch, 2013, the first species of the genus to be reported from southern China, yet not placed into any of the species groups (Golovatch 2013a, 2013b).

The present paper provides an updated review of *Antheromorpha*, based on abundant new samples from Thailand and Malaysia. In addition, type material of two species of Attems (1931, 1937) has been revised and properly redescribed. As a result, the genus is rediagnosed and a key and a distribution map are also provided.

## Material and methods

New material was taken throughout Thailand and from Malaysia between 2006 and 2015 by SP and members of the Animal Systematics Research Unit, Chulalongkorn University. Animals, both live and alcohol material, were photographed in the laboratory. Specimens were preserved in 75% ethanol and morphological investigations were carried out in the laboratory using an Olympus stereomicroscope. Scanning electron micrographs (SEM) of gonopods coated with gold were taken using a JEOL, JSM–5410 LV microscope, and the gonopods were returned to alcohol after examination. Digital images of the specimens were taken in the laboratory and assembled using the “Cell^D^” automontage software of the Olympus Soft Imaging Solution GmbH package. In addition, line drawings of gonopods were prepared. Type material of two Attemsian species of *Antheromorpha* from Thailand and Vietnam, housed in the Naturhistorisches Museum Wien, Austria (NHMW), was photographed with Dino-Eye USB Camera AM423Z, the digital images assembled using the automontage software technique and the gonopod structure redrawn. Most of the new material is kept in the Museum of Zoology, Chulalongkorn University (CUMZ), Bangkok, Thailand, except for some duplicates donated to the collections of the Natural History Museum of Denmark, University of Copenhagen, Denmark (ZMUC), the Zoological Museum, State University of Moscow, Russia (ZMUM) and the NHMW, as indicated in the text.

Collecting sites were located by GPS using the WGS84 datum.

In the synonymy sections, D stands for the original description or subsequent descriptive notes or appearance in a key, R for subsequent record or records, whereas M for a mere mention.

## Taxonomic part

### Family Paradoxosomatidae Daday, 1889 Subfamily Paradoxosomatinae Daday, 1889 Tribe Orthomorphini Brölemann, 1916

#### Genus Antheromorpha

Taxon classificationAnimaliaPolydesmidaParadoxosomatidae

Jeekel, 1968

Brachytropis Silvestri, 1896: 198 (D) (preoccupied).
Brachytropis – Attems 1937: 59 (D); Jeekel 1963: 269 (M); 1968: 57 (M).
Antheromorpha Jeekel, 1968: 57 (M).
Antheromorpha – Jeekel 1980: 71 (D); Hoffman 1980: 169 (M); Shelley et al. 2000: 84 (M); Nguyen and Sierwald 2013: 1233 (M); Golovatch 2013a: 24 (M).

##### Diagnosis.

Body medium-sized to very large (ca 19–44.5 mm long, ca 2.3–6.1 mm wide), composed of 18 podous and one apodous ring, plus telson. Paraterga from moderately to very strongly developed. Sterna without modifications. Sternal lobe or cone(s) between male coxae 4 present. Pleurosternal carinae usually well-developed. First pair of male legs without femoral adenostyles. Legs without particular modifications except for at least some ♂ legs bearing ventral brushes on tarsi, sometimes also on tibiae.

Gonopods long and rather slender; coxa slightly curved and long, with several setae distoventrally; prefemoral (= setose) part of telopodite short to very short, 1/3–1/4 as long as acropodite (= remaining part of telopodite); femorite slender to rather stout, straight to evidently curved, sometimes enlarged distally, with a strong distolateral sulcus (**s**) demarcating a “postfemoral” part; seminal groove running entirely mesally along femorite, the latter devoid of processes. Solenophore (**sph**) (= tibiotarsus) and solenomere relatively short to rather long; **sph** curved rather strongly caudad, consisting of a well-developed lamina medialis (**lm**) and a rather small lamina lateralis (**ll**); **lm** about halfway bearing a well-developed process **d**; **sph** usually bilobate to bifid, with a mesal process (**m**, or the end part of **lm**) and a ventral process (**v**, or the end part of **ll**), both supporting a long and flagelliform solenomere (**sl**).

##### Type species.

*Orthomorpha miranda* Pocock, 1895, by direct substitution.

##### Other species included.

*Antheromorpha bivittata* (Pocock, 1895), *Antheromorpha comotti* (Pocock, 1895), *Antheromorpha festiva* (Brölemann, 1896), *Antheromorpha harpaga* (Attems, 1937), *Antheromorpha mediovirgata* (Carl, 1941), *Antheromorpha minlana* (Pocock, 1895), *Antheromorpha orophila* (Carl, 1941), *Antheromorpha pardalis* (Pocock, 1895), *Antheromorpha paviei* (Brölemann, 1896), comb. n., *Antheromorpha rosea* Golovatch, 2013, *Antheromorpha uncinata* (Attems, 1931).

##### Remarks.

*Brachytropis* Silvestri, 1896, was originally established to distinguish several species of *Orthomorpha* Bollman, 1893 which occurred in Myanmar and Indochina (Jeekel 1963), with *Orthomorpha miranda* Pocock, 1895, as type species (Silvestri 1896). Because that name had been preoccupied by *Brachytropis* Fieber, 1858 (Hemiptera) (Jeekel 1963), Jeekel (1968) proposed a substitute name, *Antheromorpha*, with the same type species. In his later review of the genus, Jeekel (1980) provided its diagnosis, refined its scope, redescribed some of the constituent species and discussed their taxonomic statuses.

#### Antheromorpha miranda

Taxon classificationAnimaliaPolydesmidaParadoxosomatidae

(Pocock, 1895)

Figs 1 , 21

Orthomorpha miranda Pocock, 1895: 812 (D).
Orthomorpha miranda – Attems 1898: 327 (D); 1914: 192 (D); 1930: 132 (D); Weidner 1960: 85 (M); Jeekel 1965: 96 (M).
Orthomorpha (Orthomorpha) miranda – Attems 1936: 197 (D); 1937: 62 (D).
“Orthomorpha” miranda – Jeekel 1963: 269 (M).
Brachytropis miranda – Silvestri 1896: 198 (D).
Antheromorpha miranda – Jeekel 1968: 57 (M); 1980: 72 (D); Nguyen and Sierwald 2013: 1234 (M).
Orthomorpha bivittata Pocock, 1895: 814 (D), **syn. n.**
Orthomorpha bivittata – Attems 1898: 327 (D); 1914: 192 (D); 1930: 132 (D); Attems 1936: 204 (M); 1937: 93 (M).
“Orthomorpha” bivittata – Jeekel 1963: 269 (M).
Antheromorpha bivittata – Jeekel 1968: 57 (M); 1980: 81 (D); Nguyen and Sierwald 2013: 1234 (M).
Orthomorpha melanopleuris Pocock, 1895: 813 (D), **syn. n.**
Orthomorpha melanopleuris – Attems 1898: 337 (D); 1914: 192 (D); 1930: 132 (D); 1936: 205 (M); 1937: 94 (M); Weidener 1960: 85 (M); Jeekel 1965: 96 (M).
“Orthomorpha” melanopleuris Jeekel, 1963: 269 (M).
Antheromorpha melanopleuris – Jeekel 1968: 57 (M); 1980: 77 (D); Nguyen and Sierwald 2013: 1235 (M).

##### Remarks.

This species was described from Yangon (Rangoon) (the type locality); Tharrwaddy, Bago Division; Palon in Pegu (state/region); Thigian, upper Irrawaddy and Minhla, Myanmar (Pocock 1895). The quite large material, of which only the specimens coming from Rangoon should be considered as syntypes, because they were designated as *Types* by Pocock (1895) in the original description, is currently shared between the collections of the Natural History Museum in London, UK, the Museo Civico di Storia naturale in Genova, Italy (Jeekel 1980) and the Zoologisches Staatsinstitut und Zoologisches Museum in Hamburg, Germany (Weidner 1960). Jeekel (1980) provided a sufficiently detailed redescription of this species, based on 3 ♂ and 1 ♀ from Palon in Pegu, leg. L. Fea and 1 ♀ from Thigian, upper Irrawaddy, leg. L. Fea, mistakenly designating them as paralectotypes (= paratypes) pending the selection of a lectotype housed in the London Museum. For the time being the concept of *Antheromorpha miranda* remains based on that actually non-type material, whereas the true type series from Rangoon must be revised to finally verify the species identity, as well as to reconfirm the two new synonymies.

In addition, according to H. Enghoff (in litt.), the ZMUC collection contains a sample (3 ♂, 2 ♀, one of the males mounted on an insect pin) labelled “*Orthomorpha Miranda* Poc. // Palon // Birma Fea”. There can be no doubt this material was once received from Pocock himself.

Based solely on Jeekel’s (1980) revision of the ♀ types of *Antheromorpha bivittata* (Pocock, 1895), the ♀ lectotype and 1 ♀ paralectotype from Shenmaga, Myanmar (Pocock 1895) and of *Antheromorpha melanopleuris* (Pocock, 1895), also the ♀ lectotype and 1 ♀ paralectotype from Teinzo on the Moolay River (Pocock 1895), as well as of the non-type ♂ from Minhla, Myanmar which Pocock (1895) provisionally identified as belonging to *Antheromorpha miranda*, we venture to synonymize *Antheromorpha bivittata* and *Antheromorpha melanopleuris* with *Antheromorpha miranda*, both syn. n. In this respect we follow Jeekel (1980) who also emphasized their close resemblance to one another as regards their colour patterns and somatic characters, even though *Antheromorpha bivittata* and *Antheromorpha melanopleuris* were both based on ♀ material alone.

**Figure 1.**
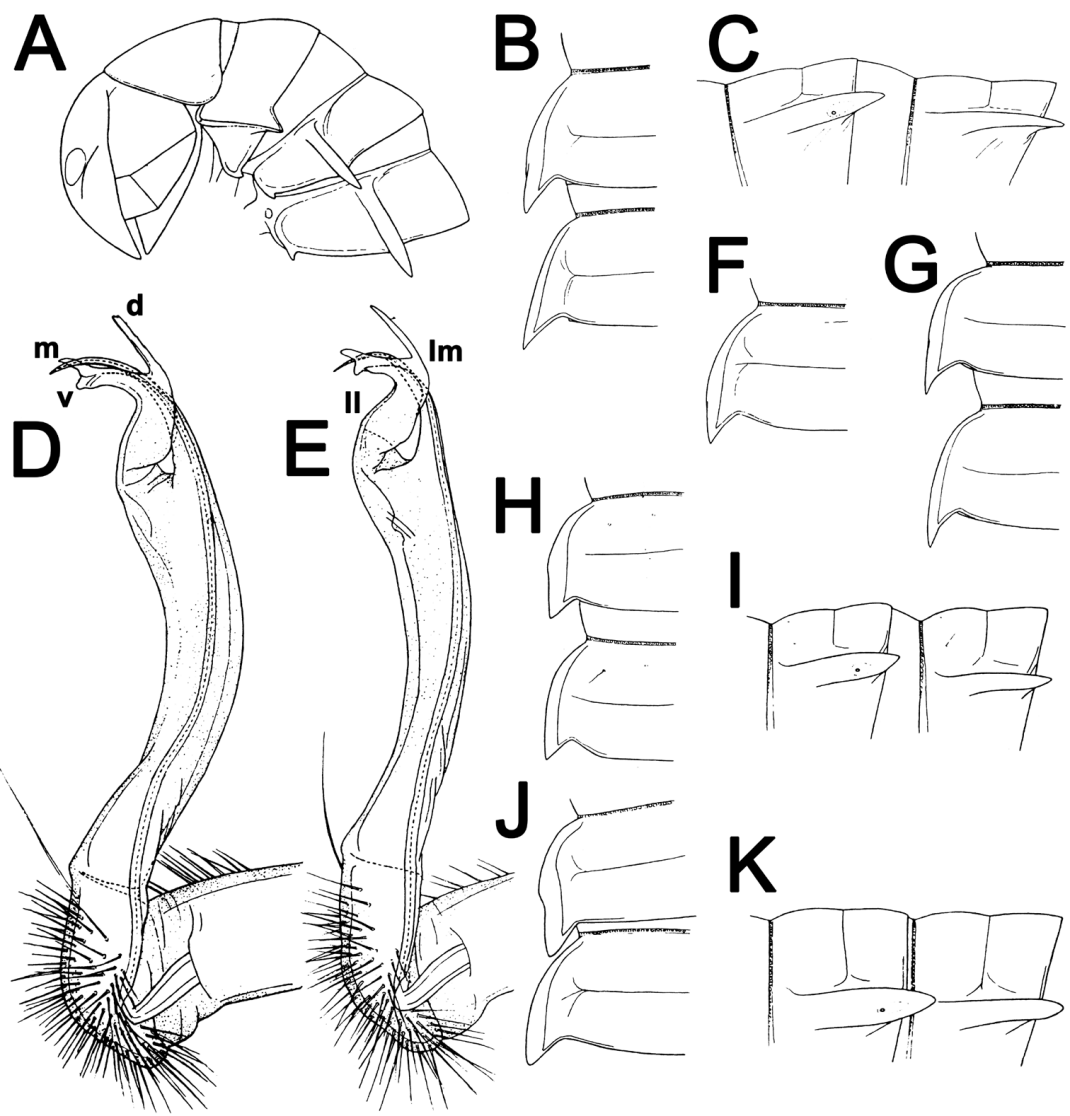
*Antheromorpha miranda* (Pocock, 1895), ♂ (**A–D**), ♀ (**F**) non-type material from Palon in Pegu, ♂ non-type material from Minhla (**E, G**), ♀ lectotype of *Orthomorpha bivittata* Pocock, 1895 (**H, I**), ♀ lectotype of *Orthomorpha melanopleuris* Pocock, 1895 (**J, K**). **A** anterior part of body, lateral view **B, C, G, H, I , J, K** segments 10 and 11, dorsal, lateral, dorsal, dorsal, lateral, dorsal and lateral views, respectively **D, E** right gonopod, mesal view **F** segment 10, dorsal view (after Jeekel 1980). No scale bar.

#### Antheromorpha bistriata

Taxon classificationAnimaliaPolydesmidaParadoxosomatidae

(Pocock, 1895)

Figs 2A–C , 21

Orthomorpha bistriata Pocock, 1895: 814 (D).
Orthomorpha bistriata – Attems 1898: 327 (D); 1914: 237 (M); 1936: 204 (M); 1937: 93 (M); Jeekel 1965: 96 (M).
“Orthomorpha” bistriata – Jeekel 1963: 269 (M).
Antheromorpha bistriata – Jeekel 1968: 57 (M).
Antheromorpha bistriata – Jeekel 1980: 79 (D); Nguyen and Sierwald 2013: 1234 (M).

##### Remark.

This species was described from Bhamo, Myanmar (Pocock 1895), redescribed by Jeekel (1980) in due detail from the ♂ holotype which is deposited in the Genova Museum, Italy.

**Figure 2.**
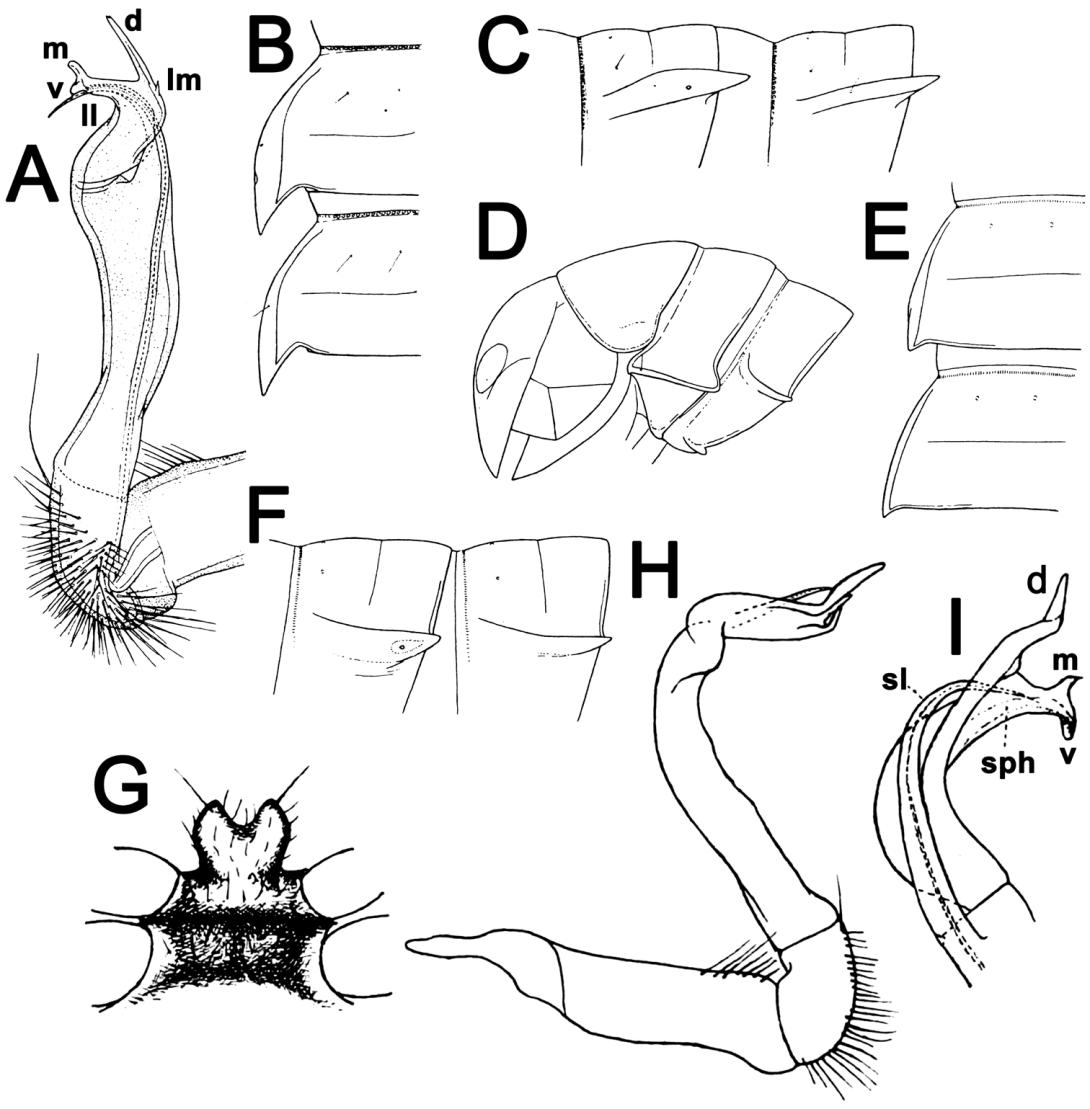
*Antheromorpha bistriata* (Pocock, 1895), ♂ holotype (**A–C**); *Antheromorpha comotti* (Pocock, 1895), ♀ holotype of *Orthomorpha comotti* Pocock, 1895 (**D–F**), ♂ holotype of Orthomorpha (Orthomorpha) orophila Carl, 1941 (**G–I**). **A, H, I** right gonopod, mesal, lateral and submesal views, respectively **B, C, E, F** segments 10 and 11, dorsal, lateral, dorsal and lateral views, respectively **D** anterior part of body, lateral view **G** sternal cones between coxae 4, subcaudal view view (after Carl 1941; Jeekel 1980). No scale bar.

#### Antheromorpha comotti

Taxon classificationAnimaliaPolydesmidaParadoxosomatidae

(Pocock, 1895)

Figs 2D–I , 21

Orthomorpha comotti Pocock, 1895: 814 (D).
Orthomorpha Comotti – Attems 1898: 327 (D); 1914: 192 (D).
Orthomorpha comotti – Attems 1898: 338 (M); 1930: 132 (D); 1936: 204 (M); 1937: 93 (M).
“Orthomorpha” comotti – Jeekel 1963: 269 (M).
Antheromorpha comotti – Jeekel 1968: 57 (M); 1980: 83 (D); Nguyen and Sierwald 2013: 1234 (M).
Orthomorpha (Orthomorpha) orophila Carl, 1941: 361 (D), **syn. n.**
“Orthomorpha” orophila – Jeekel 1963: 269 (M).
Antheromorpha orophila – Jeekel 1968: 57 (M); 1980: 85 (M); Nguyen and Sierwald 2013: 1235 (M).

##### Remarks.

This species was described and still remains known only from Minhla, Myanmar (Pocock 1895), redescribed in due detail from the ♀ holotype (now in the Genova Museum, Italy) by Jeekel (1980). Jeekel found this species not only being very similar to *Antheromorpha orophila* (Carl, 1941), which Carl (1941) had described from the northern Chin Hills, Myanmar, but he also suggested, albeit not formalized, their synonymy. Based on Jeekel’s (1980) redescription and opinion, we venture to formally synonymize *Antheromorpha orophila* under *Antheromorpha comotti*, syn. n. The syntypes (1 ♂, 1 ♀) of *Antheromorpha orophila* are in the London Museum, UK (Carl 1941).

#### Antheromorpha mediovirgata

Taxon classificationAnimaliaPolydesmidaParadoxosomatidae

(Carl, 1941)

Figs 3A, B , 21

Orthomorpha (Orthomorpha) mediovirgata Carl, 1941: 364 (D).
“Orthomorpha” mediovirgata – Jeekel 1963: 269 (M).
Antheromorpha mediovirgata – Jeekel 1968: 57 (M); 1980: 85 (M); Nguyen and Sierwald 2013: 1235 (M).

##### Remark.

This species was described and still remains known only from the northern Chin Hills, Myanmar (Carl 1941). The ♂ holotype of *Antheromorpha mediovirgata* is in the London Museum, UK (Carl 1941).

**Figure 3.**
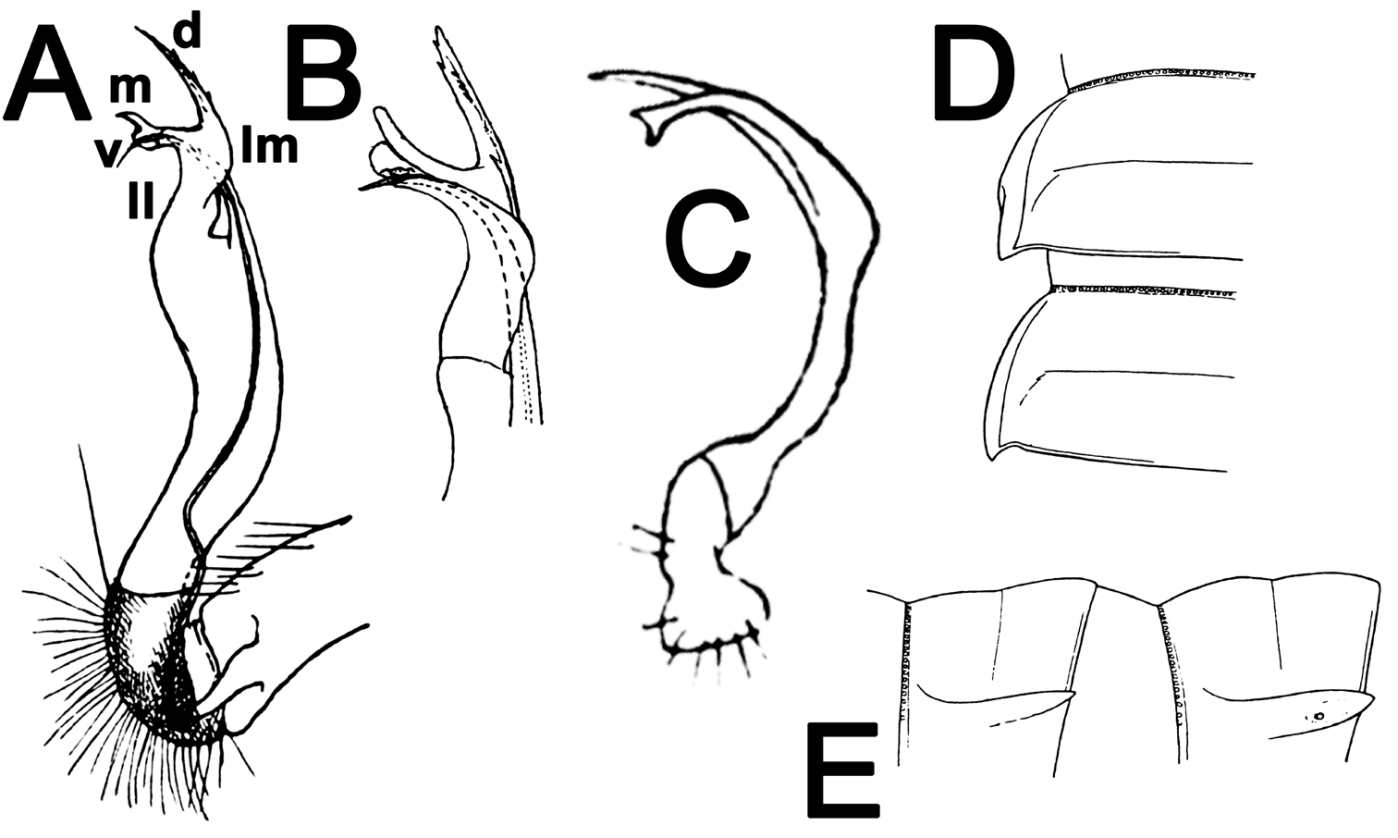
*Antheromorpha mediovirgata* (Carl, 1941), ♂ holotype (**A, B**); *Antheromorpha minlana* (Pocock, 1895), ♂ holotype (**C**); *Antheromorpha pardalis* (Pocock, 1895), ♀ holotype (**D, E**). **A, B** right gonopod, mesal and lateral views, respectively **C** left gonopod, mesal view **D, E** segments 10 and 11, dorsal and lateral views, respectively (after Pocock 1895; Carl 1941; Jeekel 1980). No scale bar.

#### Antheromorpha minlana

Taxon classificationAnimaliaPolydesmidaParadoxosomatidae

(Pocock, 1895)

Figs 3C , 21

Orthomorpha minlana Pocock, 1895: 816 (D).
Orthomorpha minlana – Attems 1898: 327 (M); 1936: 197 (D); Weidner 1960: 85 (M).
Orthomorpha (Orthomorpha) minlana – Attems 1936: 199 (M); 1937: 62 (D).
“Orthomorpha” minlana – Jeekel 1963: 269 (M).
Orthomorpha miuhlana (sic!) – Attems 1914: 193 (D).
Antheromorpha minlana – Jeekel 1968: 57 (M); 1980: 85 (M); Nguyen and Sierwald 2013: 1235 (M).

##### Remark.

This species was described and still remains known only from Minhla, Tharrawaddy District, Myanmar (Pocock 1895).

An indefinite number of ♂ and ♀ syntypes of *Antheromorpha minlana* must be deposited in the London Museum, UK (Pocock 1895). According to H. Enghoff (in litt.), the ZMUC collection contains a sample (1 ♂, 1 ♀, both mounted on insect pins) labelled “*Orthomorpha minhlana* Poc. // ex typ. //Minhla // Birma fea”.

#### Antheromorpha pardalis

Taxon classificationAnimaliaPolydesmidaParadoxosomatidae

(Pocock, 1895)

Figs 3D, E , 21

Orthomorpha pardalis Pocock, 1895: 815 (D).
Orthomorpha pardalis – Attems 1898: 327 (D); 1914: 192 (D); 1930: 132 (D); 1936: 205 (M); 1937: 94 (M).
“Orthomorpha” pardalis – Jeekel 1963: 269 (M).
Antheromorpha pardalis – Jeekel 1968: 57 (M); Jeekel 1980: 82 (D); Nguyen and Sierwald 2013: 1235 (M).

##### Remark.

This species was described and still remains known only from a single ♀, the holotype which comes from Palon in Pegu, Myanmar (Pocock 1895) and is kept in the Genova Museum, Italy (Jeekel 1980). The species is similar to *Antheromorpha miranda* (Pocock, 1895), but has a different colour pattern of the metaterga, the latter showing yellowish paramedian spots in front of the transverse sulcus (versus yellowish paramedian stripes), coupled with the sulcus starting with segment 2 (versus segment 5). Since the colour pattern is one of the most important taxonomic characters for species discrimination in the genus, *Antheromorpha pardalis* for the time being is regarded as a separate species. However, only the discovery of topotypical ♂ specimens can provide decisive information concerning the identity of this species (Jeekel 1980).

#### Antheromorpha paviei

Taxon classificationAnimaliaPolydesmidaParadoxosomatidae

(Brölemann, 1896) comb. n.

Figs 4 , 5 , 6 , 21

Orthomorpha Paviei Brölemann, 1896: 1 (D).
Orthomorpha Paviei – Brölemann 1904: 8 (D).
?Prionopeltis Paviei – Attems 1914: 204 (M).
Pratinus Paviei – Attems 1937: 122 (M).
Orthomorpha paviei – Jeekel 1963: 265 (M); 1964: 359 (M); 1968: 56 (M); Golovatch 1998: 42 (D); Enghoff et al. 2004: 34 (M); Enghoff 2005: 97 (M); Likhitrakarn et al. 2011: 52 (D); 2014: 2 (D, R).

##### Remarks.

Redescribed based on new material from Khone Phapen Waterfall, Laos (Likhitrkarn et al. 2014), this species is distinguished by a more *Orthomorpha*-like colour patterm (only paraterga being contrasting light), yet, like a rather typical *Antheromorpha*, its solenophore tip is deeply split. It is the latter character that justifies the assignment of this species to *Antheromorpha*. The ♂ holotype of *Antheromorpha paviei* is deposited in the Paris Museum, France (Brölemann 1896, 1904).

**Figure 4.**
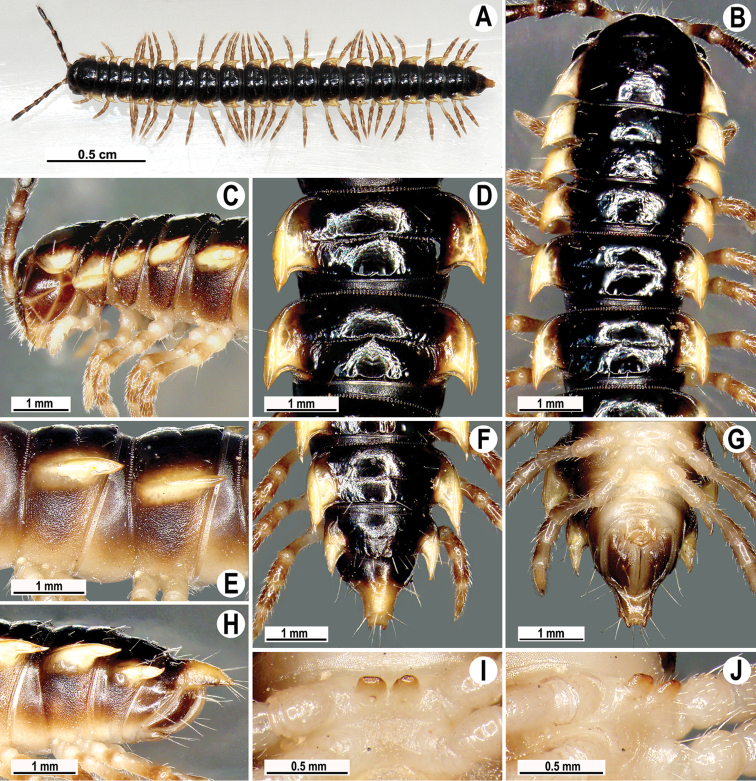
*Antheromorpha paviei* (Brölemann, 1896), ♂ from Laos. **A** habitus, live coloration **B, C** anterior part of body, dorsal and lateral views, respectively **D, E** segments 10 and 11, dorsal and lateral views, respectively **F–H** posterior part of body, dorsal, ventral and lateral views, respectively **I, J** sternal cones between coxae 4, subcaudal and sublateral views, respectively (After Likhitrakarn et al. 2014).

**Figure 5.**
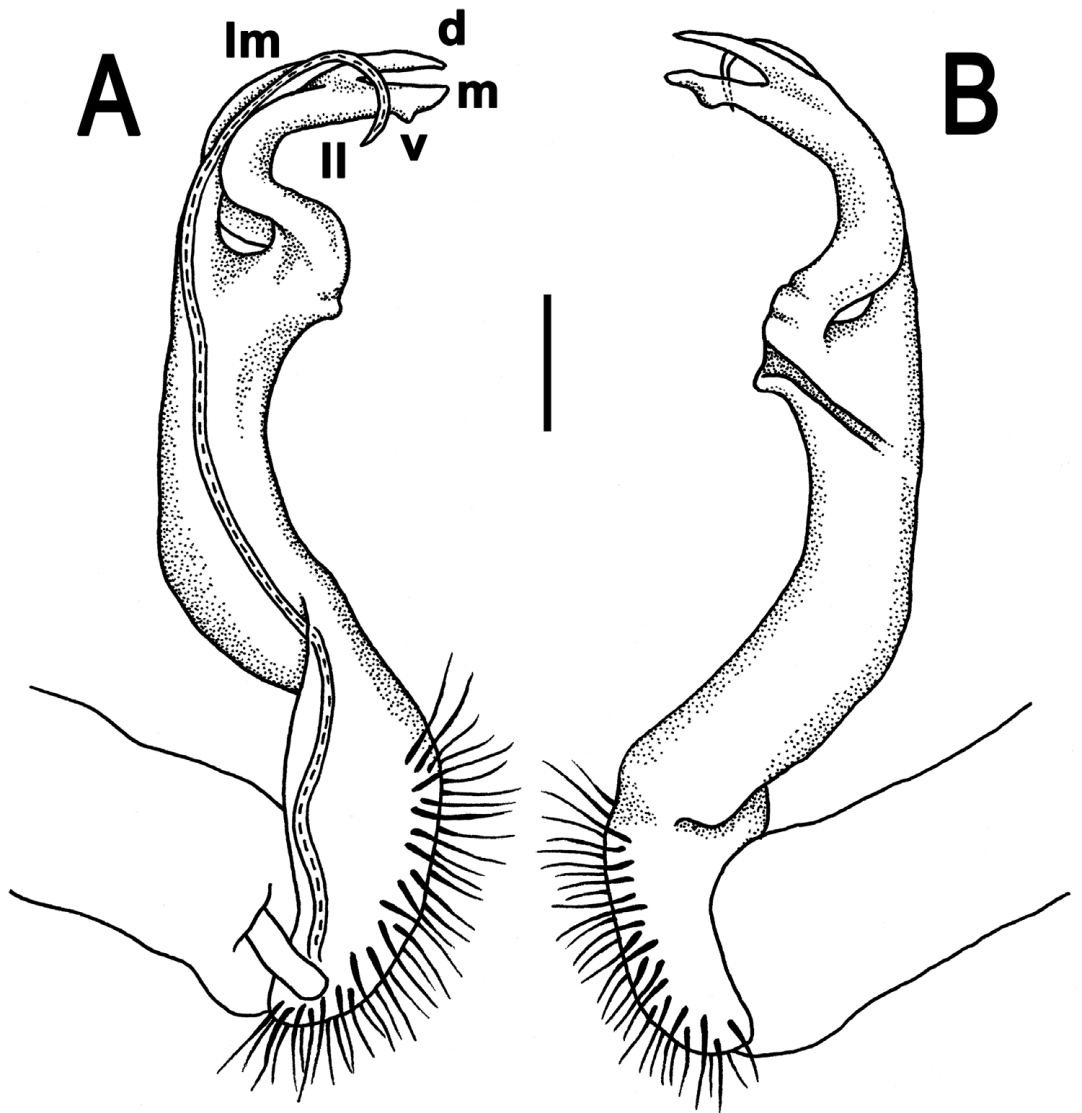
*Antheromorpha paviei* (Brölemann, 1896), ♂. **A, B** left gonopod, mesal and lateral views, respectively (After Likhitrakarn et al. 2014). Scale bar: 0.5 mm.

**Figure 6.**
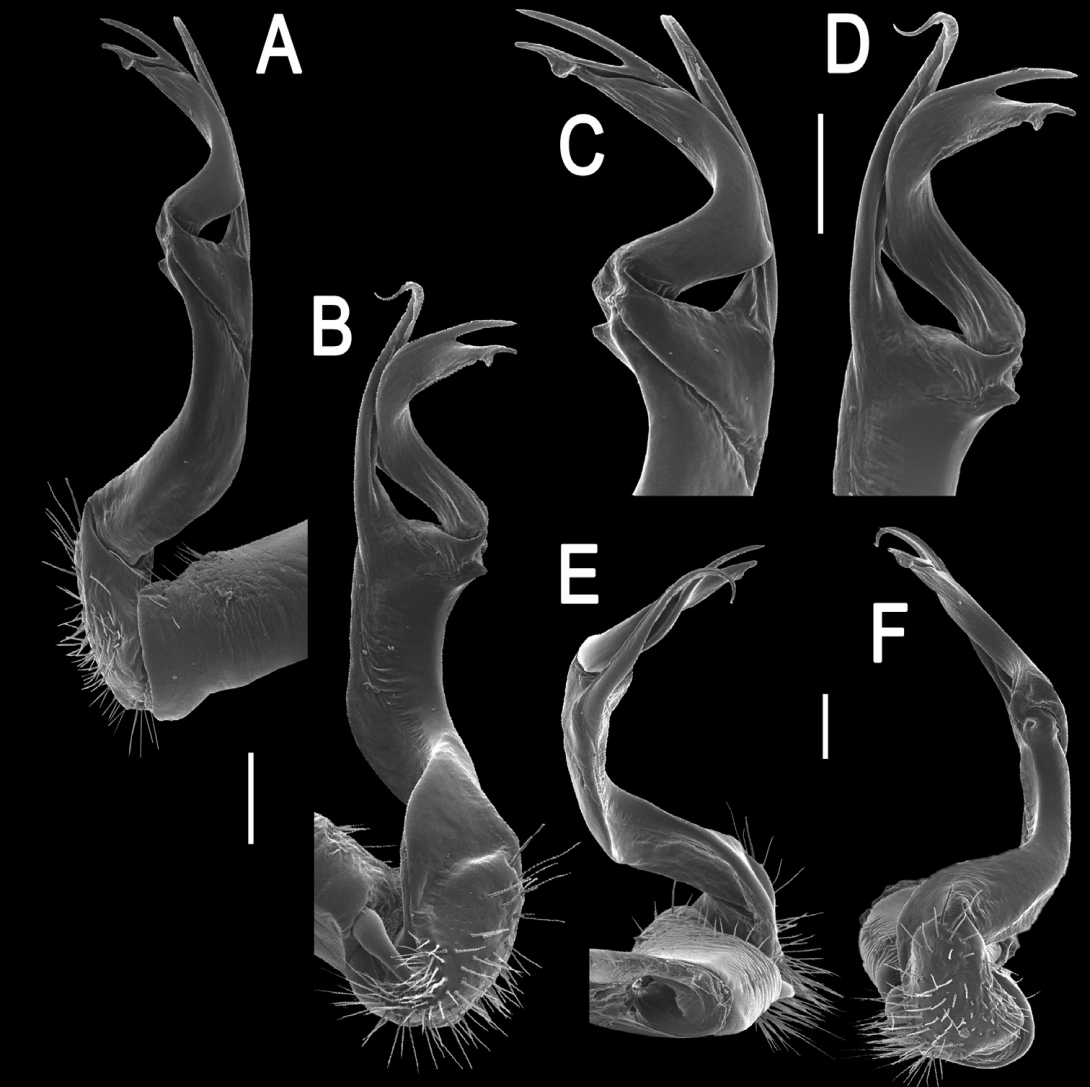
*Antheromorpha paviei* (Brölemann, 1896), ♂ from Laos, left gonopod. **A, B** lateral and mesal views, respectively **C, D** telopodite, lateral, mesal **E, F** distal part, subcaudal and suboral views, respectively (After Likhitrakarn et al. 2014). Scale bar: 0.2 mm.

#### Antheromorpha uncinata

Taxon classificationAnimaliaPolydesmidaParadoxosomatidae

(Attems, 1931)

**Figure 7.**
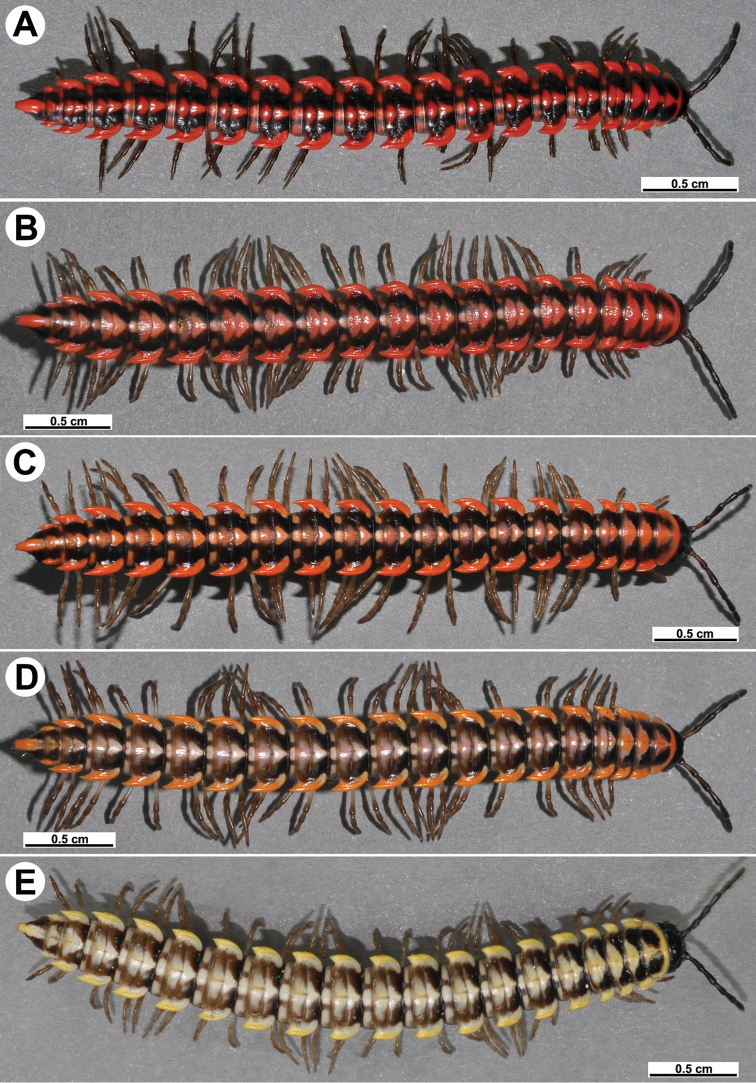
*Antheromorpha uncinata* (Attems, 1931), Habitus, live coloration (**A–E**) ♂ from Sakaerat Environmental Research Station Sakaerat Biosphere Reserves (**A**), ♂ from Tham Prakaiphet (**B**), ♂ from Srinakharin Dam (**C, D**), ♂ from Thap Sakae (**E**).

**Figure 8.**
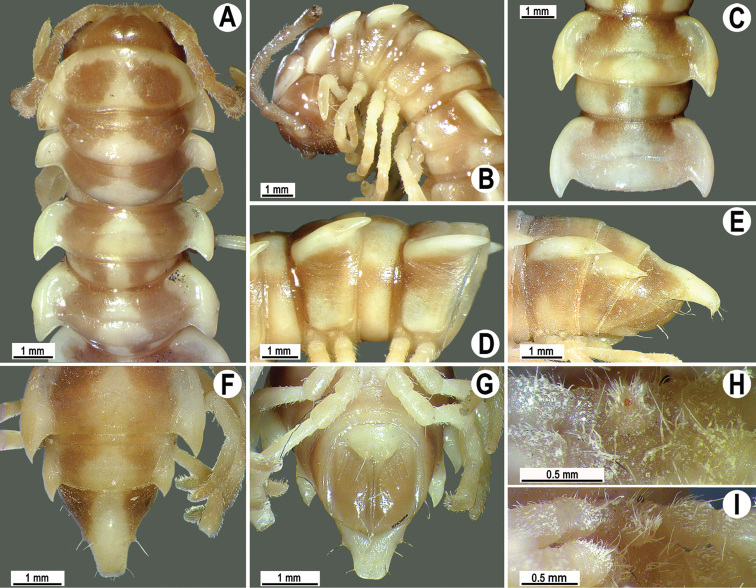
*Antheromorpha uncinata* (Attems, 1931), ♂ lectotype. **A, B** anterior part of body, dorsal and lateral views, respectively **C, D** segments 10 and 11, dorsal and lateral views, respectively **E–G** posterior part of body, lateral, dorsal and ventral views, respectively **H, I** sternal cones between coxae 4, subcaudal and sublateral views, respectively.

**Figure 9.**
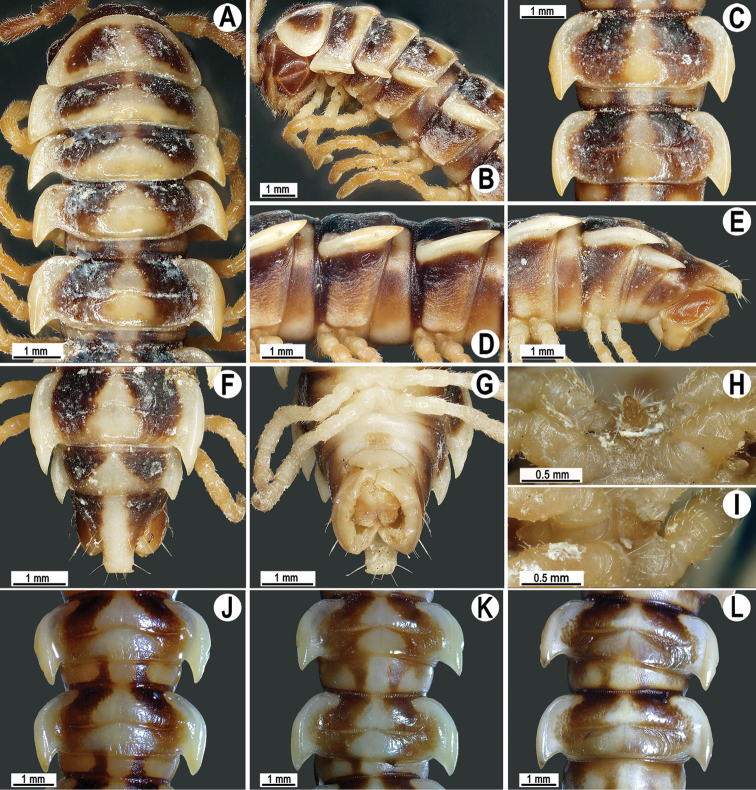
*Antheromorpha uncinata* (Attems, 1931), ♂ from Wat Tham Phromlok Khaoyai (**A–I**), ♂ from Tham Prakaiphet (**J**), ♂ from Tham Phet Phothong (**K**), male from Thap Sakae (**L**). **A, B** anterior part of body, dorsal and lateral views, respectively **C, D, J–L** segments 10 and 11, dorsal, lateral, dorsal, dorsal and dorsal views, respectively **E–G** posterior part of body, lateral, dorsal and ventral views, respectively **H, I** sternal cones between coxae 4, subcaudal and sublateral views, respectively.

**Figure 10.**
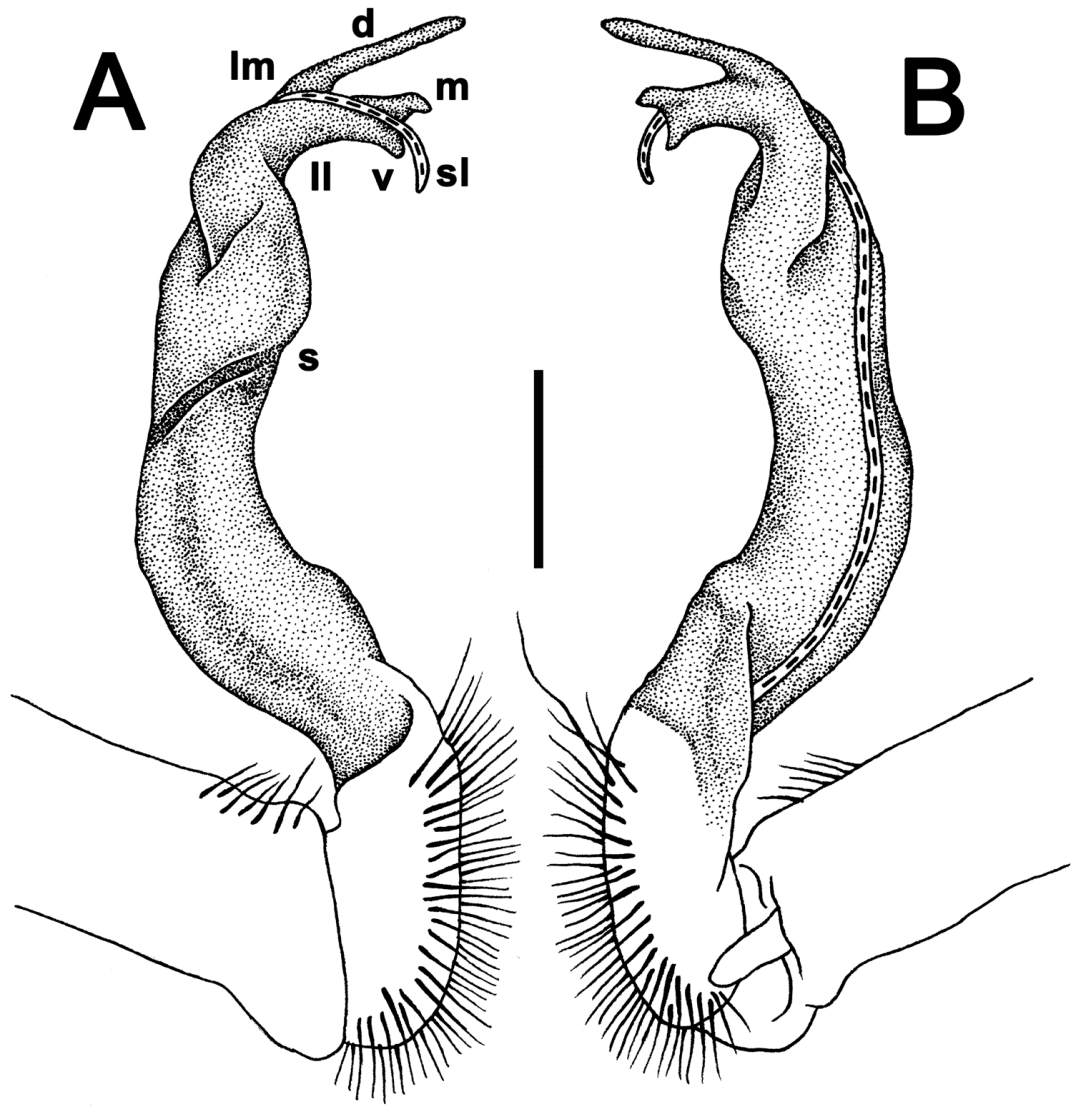
*Antheromorpha uncinata* (Attems, 1931), ♂ lectotype. **A, B** right gonopod, lateral and mesal views, respectively. Scale bar: 0.4 mm.

**Figure 11.**
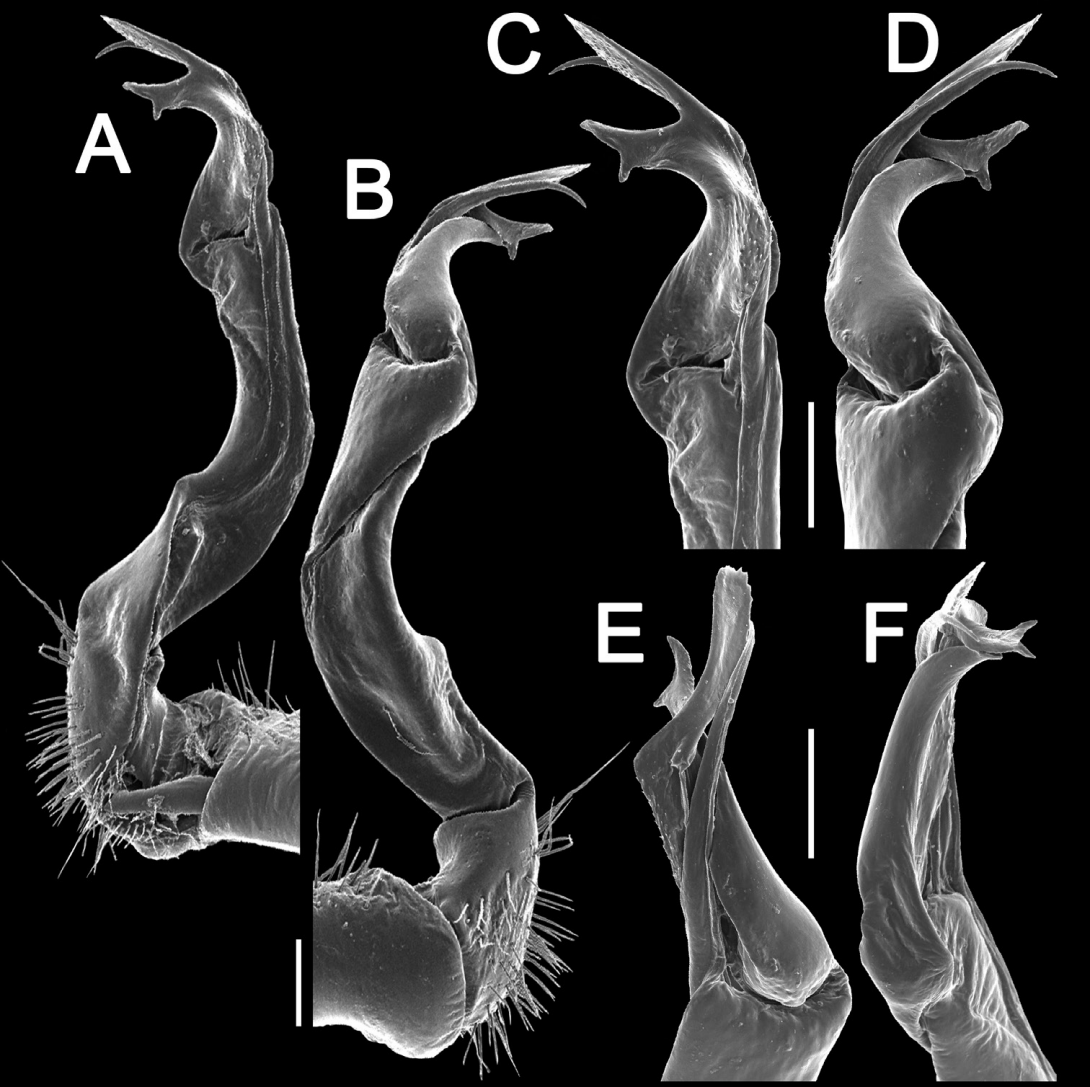
*Antheromorpha uncinata* (Attems, 1931), ♂ from Wat Tham Phromlok Khaoyai. **A, B** right gonopod, mesal and lateral views, respectively **C–F** distal part of right gonopod, mesal, lateral, subcaudal and suboral views, respectively. Scale bar: 0.2 mm.

**Figure 12.**
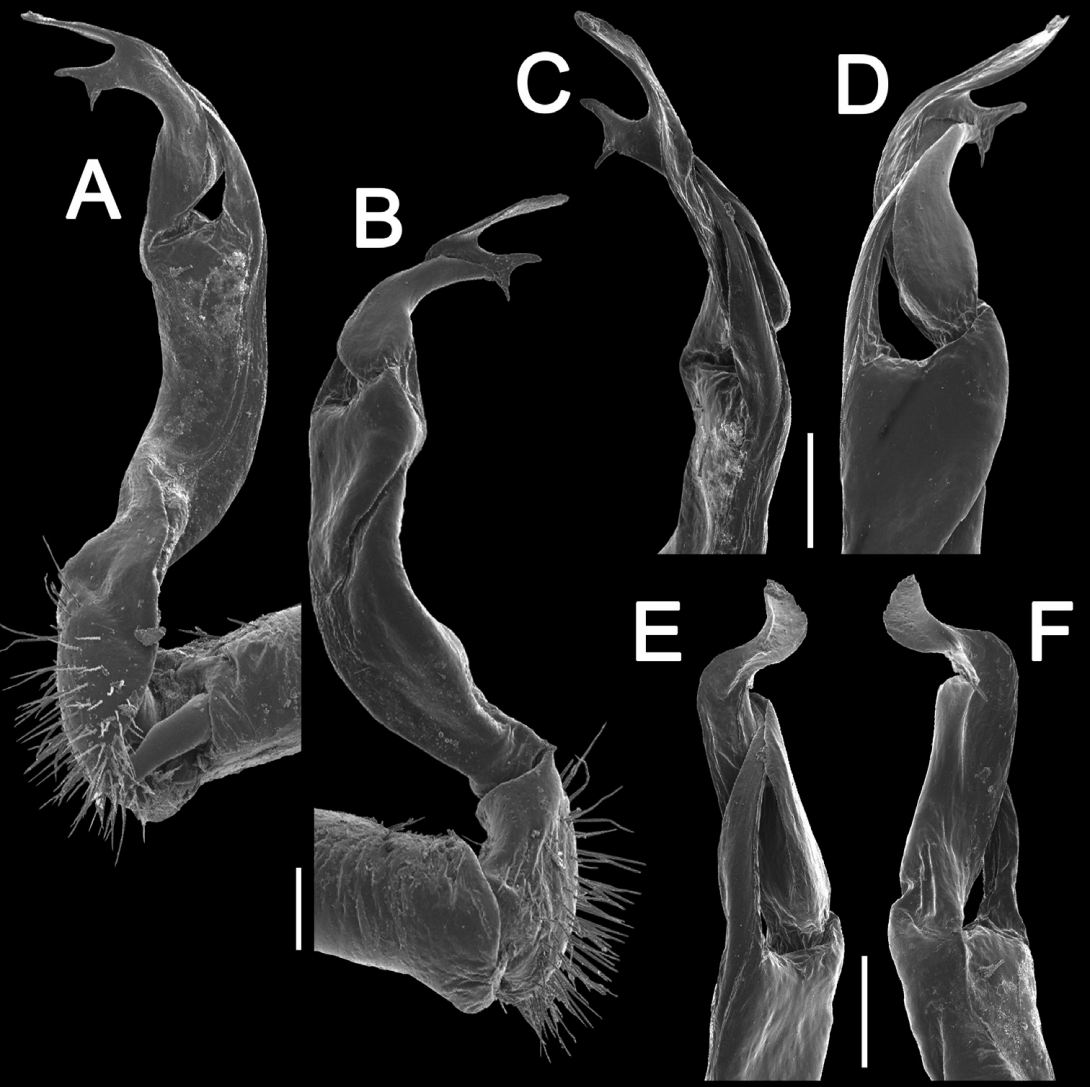
*Antheromorpha uncinata* (Attems, 1931), ♂ from Thap Sakae. **A, B** right gonopod, mesal and lateral views, respectively **C–F** distal part of right gonopod, submesal, sublateral, subcaudal and suboral views, respectively. Scale bar: 0.2 mm.

Figs 7 , 8 , 9 , 10 , 11 , 12 , 21

Orthomorpha (Orthomorpha) uncinata Attems, 1931: 117 (D).
Orthomorpha uncinata – Attems 1930: 132 (D); 1936: 197 (D).
Orthomorpha (Orthomorpha) uncinata – Attems 1936: 199 (M); 1937: 62 (D); Weidner 1960: 86 (M).
“Orthomorpha” uncinata – Jeekel 1963: 269 (M).
Antheromorpha uncinata – Jeekel 1968: 57 (M); 1980: 85 (M); Enghoff 2005: 95 (R); Nguyen and Sierwald 2013: 1235 (M).

##### Lectotype

♂ of *Orthomorpha uncinata* (NHMW-3496), Thailand, Muok Lek, 01–02.1901, leg. H. Fruhstorfer.

##### Paralectotype.

1 ♀ (NHMW-3496), same locality, together with lectotype.

Lectotype designation proposed herewith is necessary to ensure the species is based on a male.

##### Other material examined.

4 ♂, 9 ♀ (CUMZ), Thailand, Kanchanaburi Province, Sai Yok District, Sai Yoi Noi Waterfall, 14°14'14"N, 99°03'30"E, ca 150 m a.s.l., 08.05.2014, leg. P. Jirapatrasilp. 1 ♀ (CUMZ), same locality, 08.05.2010, leg. N. Likhitrakarn. 7 ♂, 4 ♀ (CUMZ), same District, Wat Tham Phromlok Khaoyai, 14°12'14"N, 99°07'57"E, ca 120 m a.s.l., 09.07.2009, leg. S. Panha, N. Likhitrakarn and C. Sutcharit. 1 ♀ (CUMZ), same locality, 29.10.2013, leg. R. Saokord. 3 ♀ (CUMZ), same District, Chong Khao Khat, 14°22'23"N, 98°55'40"E, 414 m a.s.l., 29.08.2011, leg. S. Panha, N. Likhitrakarn and C. Sutcharit. 1 ♂, 2 ♀ (CUMZ), same District, near Cave Krasae, 14°09'12"N, 99°06'37"E, ca 75 m a.s.l., 10.12.2006, leg. S. Panha and C. Sutcharit. 2 ♂ (CUMZ), same Province, Si Sawat District, Arawan Waterfall, 14°22'31"N, 99°08'39"E, ca 90 m a.s.l., 13.05.2010, leg. S. Panha, N. Likhitrakarn and C. Sutcharit. 1 ♀ (CUMZ), same District, Chaloem Rattanakosin National Park, Tham Lod Noi, 14°39'29"N, 99°18'19"E, ca 320 m a.s.l., 10.07.2006, leg. S. Panha and C. Sutcharit. 3 ♂ (CUMZ), same District, Srinakharin Dam, 14°24'09"N, 99°07'34"E, ca 220 m a.s.l., 13.05.2010, leg. S. Panha, N. Likhitrakarn and C. Sutcharit. 2 ♂, 2 ♀ (CUMZ), same Province, Nong Prue District, Wat Tham Phukung, 14°28'18"N, 99°06'34"E, ca 200 m a.s.l., 20.12.2013, leg. S. Panha, R. Saokord and C. Sutcharit. 12 ♂, 5 ♀ (CUMZ), 2 ♂, 1 ♀ (ZMUM ρ3056), 2 ♂, 1 ♀ (ZMUC), 2 ♂, 1 ♀ (NHMW), Uthai Thani Province, Ban Rai District, Tham Prakaiphet, 15°12'17"N, 99°44'01"E, ca 90 m a.s.l., 08.07.2009, leg. N. Likhitrakarn, S. Panha and C. Sutcharit. 7 ♂, 10 ♀, 1 juv. (CUMZ), same District, Wat Tham Khao Wong, 15°01'59"N, 99°27'18"E, ca 110 m a.s.l., 08.07.2009, leg. N. Likhitrakarn, S. Panha and C. Sutcharit. 10 ♂, 5 ♀, 7 juv. (CUMZ), same District, Huaykhakhaeng Country Home Resort, 15°06'02"N, 99°35'42"E, ca 210 m a.s.l., 07.06.2008, leg. N. Likhitrakarn, S. Panha and C. Sutcharit. 1 ♀ (CUMZ), same locality, 27.10.2013, leg. S. Panha, R. Saokord and C. Sutcharit. 2 ♂, 2 ♀ (CUMZ), Sa Kaeo Province, Khlong Hat District, Tham Phet Phothong, 13°24'47"N, 102°19'32"E, ca 200 m a.s.l., 28.10.2010, leg. N. Likhitrakarn, S. Panha and C. Sutcharit. 1 ♂, 2 ♀ (CUMZ), same locality, 22.05.2012, leg. R. Saokord. 1 ♂, 2 ♀ (CUMZ), same Province, Ta Phraya District, Amphoe Ta Phraya, 14°08'22"N, 102°40'11"E, ca 180 m a.s.l., 27.10.2010, leg. N. Likhitrakarn, S. Panha and C. Sutcharit. 1 ♂ (CUMZ), same Province, Wang Sombun District, Thamkhao Phrapphrueng Thong, 13°26'55"N, 102°13'02"E, ca 180 m a.s.l., 22.05.2012, leg. N. Likhitrakarn, S. Panha and C. Sutcharit. 1 ♀ (CUMZ), Loei Province, Nong Hin District, Wat Tham Pho Thi Sat, 17°05'17"N, 101°46'51"E, 405 m a.s.l., 19.10.2007, leg. S. Panha and C. Sutcharit. 1 ♂ (CUMZ), same District, Wat Tham Dok Bua, 17°03'14"N, 101°44'39"E, ca 680 m a.s.l., 12.06.2013, leg. S. Panha and C. Sutcharit. 1 ♂ (CUMZ), same District, Hin Pha Ngam Park, 17°03'02"N, 101°44'37"E, ca 680 m a.s.l., 19.10.2007, leg. S. Panha and C. Sutcharit. 2 ♂ (CUMZ), Wang Saphung District, Wat Tham Wangsaphung, 17°19'38"N, 101°39'59"E, ca 275 m a.s.l., 18.10.2007, leg. S. Panha and C. Sutcharit. 1 ♂, 2 ♀ (CUMZ), Chiang Mai Province, Mae Rim District, near Mae Rim city, 18°54'23"N, 98°54'14.76"E, ca 340 m a.s.l., 17.09.2015, leg. N. Nantarat. 1 ♀ (CUMZ), Chiang Rai Province, Mueang Chiang Rai District, Pang Rimkorn, 19°50'51"N, 99°40'04"E, 485 m a.s.l., 10.07.2006, leg. S. Panha and C. Sutcharit. 2 ♂, 2 ♀, 1 juv. (CUMZ), Lopburi Province, Phatthana Nikhom District, Wat Dilang, 14°56'15"N, 100°53'46"E, ca 85 m a.s.l., 11.07.2008, leg. S. Panha and C. Sutcharit. 1 ♂, 1 ♀ (CUMZ), Phetchabun Province, Bueng Sam Phan District, Ban Phanom Phet, 15°46'56"N, 100°49'37"E, ca 100 m a.s.l., 10.04.2007, leg. S. Panha and C. Sutcharit. 5 juv. (CUMZ), same Province, Nam Nao District, Nam Nao National Park, 16°45'26"N, 101°33'41"E, ca 925 m a.s.l., 19.06.2014, leg. S. Noommeechai. 1 ♀ (CUMZ), Saraburi Province, Kaeng Khoi District, Siharatdechochai, 14°41'05"N, 101°03'17"E, ca 60 m a.s.l., 19.09.2009, leg. S. Panha and C. Sutcharit. 1 ♀ (CUMZ), Ratchaburi Province, Mueang Ratchaburi District, Wat Tham Khaobin, 13°35'35"N, 99°40'03"E, ca 50 m a.s.l., 30.10.2013, leg. S. Panha and C. Sutcharit. 1 ♀ (CUMZ), Sukhothai Province, Si Samrong District, Wat Tham Rakhang, 17°10'02"N, 99°33'29"E, ca 200 m a.s.l., 19.09.2009, leg. S. Panha and C. Sutcharit. 4 ♂, 1 ♀ (CUMZ), Nakhon Sawan Province, Mueang Nakhon Sawan District, Wat Mano, 15°48'41"N, 99°54'55"E, ca 90 m a.s.l., 29.05.2009, leg. S. Panha and C. Sutcharit. 1 ♂ (CUMZ), same Province, Takhli District, Wat Thampratun Temple, 15°14'07"N, 100°22'11"E, ca 30 m a.s.l., 27.10.2015, leg. N. Likhitrakarn and C. Sutcharit. 1 ♂, 1 ♀ (CUMZ), Nakhon Ratchasima Province, Wang Nam Khiao District, Sakaerat Environmental Research Station Sakaerat Biosphere Reserves, 14°30'42"N, 101°56'35"E, ca 340 m a.s.l., 24.04.2009, leg. N. Likhitrakarn, S. Panha and C. Sutcharit. 2 ♀, 2 juv. (CUMZ), same locality, 03.08.2013, leg. R. Saokord. 2 ♂, 2 ♀ (CUMZ), same Province, Pak Chong District, Khao Rup Chang, 14°31'33"N, 101°21'36"E, ca 415 m a.s.l., 24.04.2009, leg. N. Likhitrakarn, S. Panha and C. Sutcharit. 2 ♂, 3 ♀ (CUMZ), Prachuap Khiri Khan Province, Thap Sakae District, Thap Sakae, 11°33'57"N, 99°32'56"E, 85 m a.s.l., 31.08.2011, leg. N. Likhitrakarn, S. Panha and C. Sutcharit. 1 ♂, 1 ♀ (CUMZ), same Province, Bang Saphan District, Tham Khao Ma Rong, 11°12'20"N, 99°29'44"E, 7 m a.s.l., 12.10.2008, leg. S. Panha and C. Sutcharit. 3 ♀ (CUMZ), same locality, 22.05.2010, leg. N. Likhitrakarn, S. Panha and C. Sutcharit. 1 ♂ (CUMZ), same Province, Hua Hin District, Kaeng Krachan, 12°45'32"N, 99°32'59"E, ca 570 m a.s.l., 24.01.2012, leg. N. Likhitrakarn, S. Panha and C. Sutcharit. 1 ♂ (CUMZ), same Province, Kui Buri District, Ban Yang Chum, 12°05'33"N 99°42'59"E, ca 60 m a.s.l., 07.08.2014, leg. N. Likhitrakarn, S. Panha and C. Sutcharit. 1 ♂ (CUMZ), same Province, Kui Buri District, Kui Buri National Park, 12°03'05"N 99°37'24"E, 150 m a.s.l., 15.03.2010, leg. N. Likhitrakarn, S. Panha and C. Sutcharit.

##### Redescription.

Length 30.0–42.5 (♂) or 34.0–44.5 mm (♀), width of midbody pro- and metazonae 2.6–4.0 and 2.9–4.4 mm (♂), 3.9–5.3 and 4.4–6.1 mm (♀), respectively.

Coloration of live animals red, orange to yellow (Fig. 7), with blackish to dark brown parallel bands on metaterga and prozonae; head and antennae blackish, legs dark to light brown (Fig. 7); coloration in alcohol, after a long term preservation, faded to pale yellowish (Figs 8, 9), the parallel bands faded to brownish to pale brown, head and antennae light brown to dark brown, legs and venter light yellowish to pale yellowish (Figs 8, 9).

Clypeolabral region densely, vertex sparsely setose; epicranial suture distinct. Antennae long (Figs 7, 8A, B, 9A), extending behind metaterga 3 when stretched dorsally (♂, ♀). In width, head < collum < segment 4 < 3 < 2 < 5–17 (♂) or head < collum < segment 2 < 4 < 3 < 5–17 (♀), gently and gradually tapering thereafter. Collum with three transverse rows of setae: 4+4 in anterior, 2+2 in intermediate and 3+3 in posterior row; caudal corner of paraterga rounded, declined, not extending behind rear tergal margin (Figs 8A, 9A).

Tegument smooth and shining, prozonae finely shagreened, metaterga leathery and faintly rugulose, surface below paraterga finely microgranulate. Postcollum metaterga with two transverse rows of setae traceable at least as insertion points when setae broken off: 2+2 in anterior (pre-sulcus) and 3+3 in posterior (post-sulcus) row. Tergal setae long, strong, slender, about 1/3 of metatergal length. Axial line visible only on metaterga. Paraterga very strongly developed (Figs 8A–G, 9A–G, J–L), especially well so in ♂, mostly upturned, subhorizontal, all lying below dorsum, set at about upper 1/3 of midbody height, caudal corner narrowly rounded to pointed, increasingly strongly spiniform and produced behind rear tergal margin, best developed and slightly curved mesad on segments 15–19; in lateral view, paraterga modestly enlarged on pore-bearing segments, thinner on poreless ones. Calluses delimited only by a dorsal sulcus. Paraterga 2 broad, anterior edge angular, lateral edge with one larger and two smaller, but evident incisions in anterior 1/3; posterior edge well concave (Fig. 9A). Following paraterga with anterior edge broadly rounded, bordered and fused to callus, lateral edge without incisions, caudal corners extending behind tergal margin, posterior edge oblique to clearly concave, especially well so in segments 16–19 (Figs 8F, 9F). Ozopores evident, lateral, lying in an ovoid groove at about 1/3 of metatergite’s length in front of caudal corner. Transverse sulcus usually distinct (Figs 8A, C, F, 9A, C, F, J–L), complete on metaterga 5–18, shallow, not reaching bases of paraterga, very faintly beaded at bottom, incomplete and nearly wanting on segments 4 and 19. Stricture between pro- and metazonae wide, rather deep, beaded at bottom down to base of paraterga (Figs 8A–F, 9A–D, J–L). Pleurosternal carinae complete crests with a sharp caudal tooth on segments 2–4, thereafter crests bulged anteriorly and with a small, sharp, caudal tooth on segments 5–9, a very small denticle on segments 10–15 (♂) (Figs 8B, D, E, 9B, D, E) or crests bulged anteriorly and with a small, sharp, caudal tooth on segments 5–10, thereafter a very small denticle on segments 11–14 (♀). Epiproct (Figs 8E–G, 9E–G) conical, flattened dorsoventrally, with two evident, caudoventrally curved, apical papillae; tip subtruncate; pre-apical papillae small, but visible, lying rather close to tip. Hypoproct roundly subtrapeziform to subtriangular, setiferous knobs at caudal edge evident and well-separated.

Sterna sparsely setose, without modifications; a large, central, setose cone between ♂ coxae 4 (Figs 8H, J, 9H, J). No conspicuous ridge in front of gonopod aperture. Legs moderately long and slender, midbody ones ca 1.2–1.4 (♂) or 0.8–0.9 (♀) times as long as body height, prefemora without modifications, ♂ tarsal brushes present until legs of segment 17.

Gonopods (Figs 10–12) with femorite about 3 times as long as prefemoral (= strongly setose) part. Femorite rather stout and long, strongly curved, postfemoral portion demarcated by an oblique lateral sulcus; tip of solenophore (**sph**) rather deeply bifid; process **d** slender, rounded to nearly pointed; process **m** rounded, longer than process **v**.

##### Remarks.

This species was described from Muok Lek, Thailand (Attems 1931). Enghoff (2005), based on ZMUC material, added another four localities: Kamphaeng Phet Province; Sitang, Northeast Thailand; Phu Kradung; Phu Kugio, field on way to communist camp, Chayaphum Province. We revised Attems’ type specimens, both in NHMW, and herewith designate a lectotype to ensure that the name-bearing specimen is a complete ♂. In most of their characters, the new samples are very similar to the type series except for body size and the shape of paraterga. In one and the same population, variation in the shape of paraterga is often observed, these ranging from more to less convex laterally and more or less strongly drawn caudad behind the rear tergal margin (Figs 8A, C, F, 9A, C, F, J–L). In addition, colour variations can be seen, the body being mostly red (prevailing), orange or yellow, with all possible intergradations (Fig. 7). It is noteworthy that only adults show colour variations, whereas juveniles are colourless. At Pang Rimkorn, Chiang Rai Province, *Antheromorpha uncinata* has been observed as showing swarming behaviour.

#### Antheromorpha rosea

Taxon classificationAnimaliaPolydesmidaParadoxosomatidae

Golovatch, 2013

**Figure 13.**
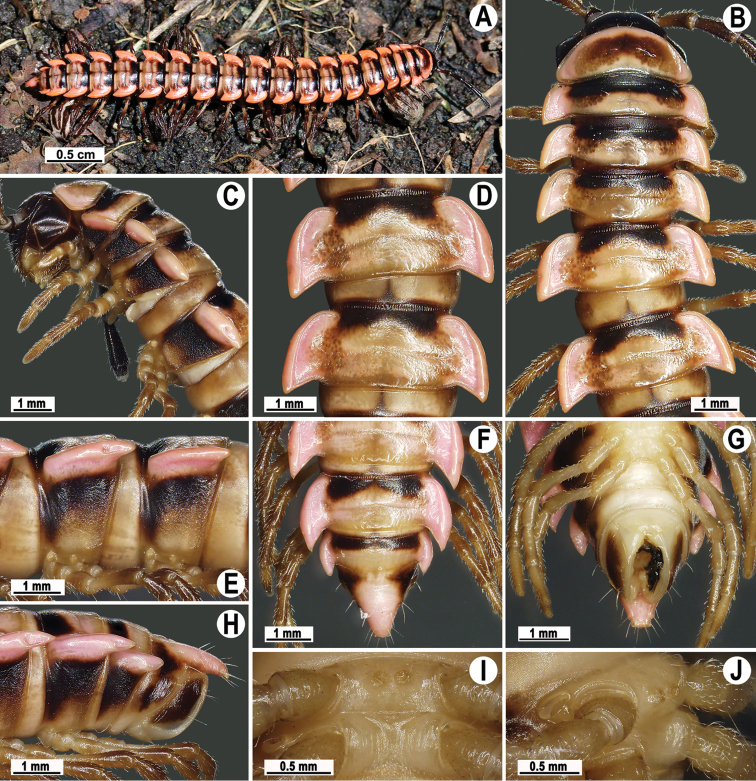
*Antheromorpha rosea* Golovatch, 2013, ♂ from Queen Sirikit Botanic Garden. **A** habitus, live coloration **B, C** anterior part of body, dorsal and lateral views, respectively **D, E** segments 10 and 11, dorsal and lateral views, respectively **F–H** posterior part of body, lateral, dorsal and ventral views, respectively **I, J** sternal cones between coxae 4, subcaudal and sublateral views, respectively.

**Figure 14.**
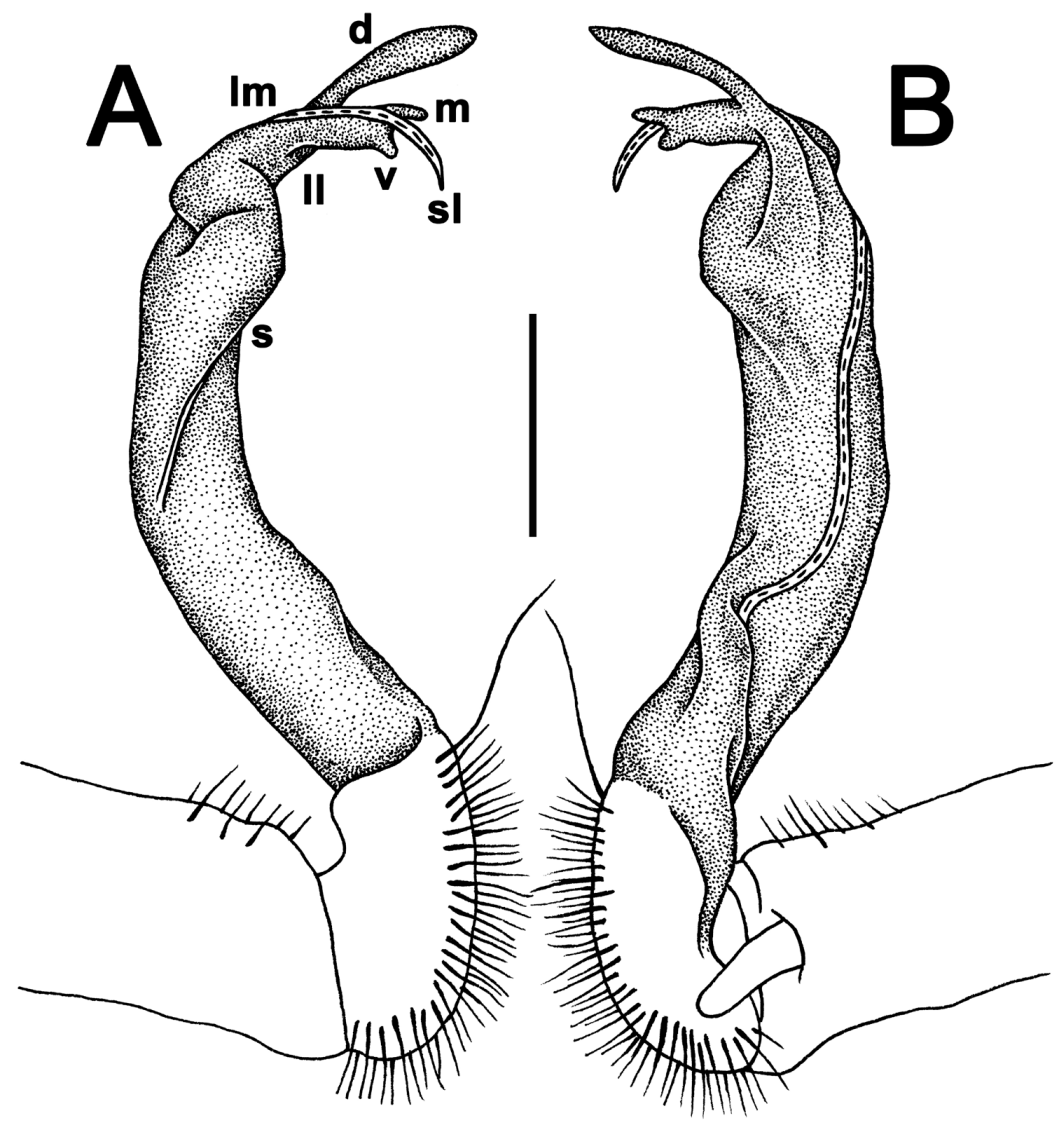
*Antheromorpha rosea* Golovatch, 2013, ♂ from Queen Sirikit Botanic Garden. **A, B** right gonopod, lateral and mesal views, respectively. Scale bar: 0.4 mm.

**Figure 15.**
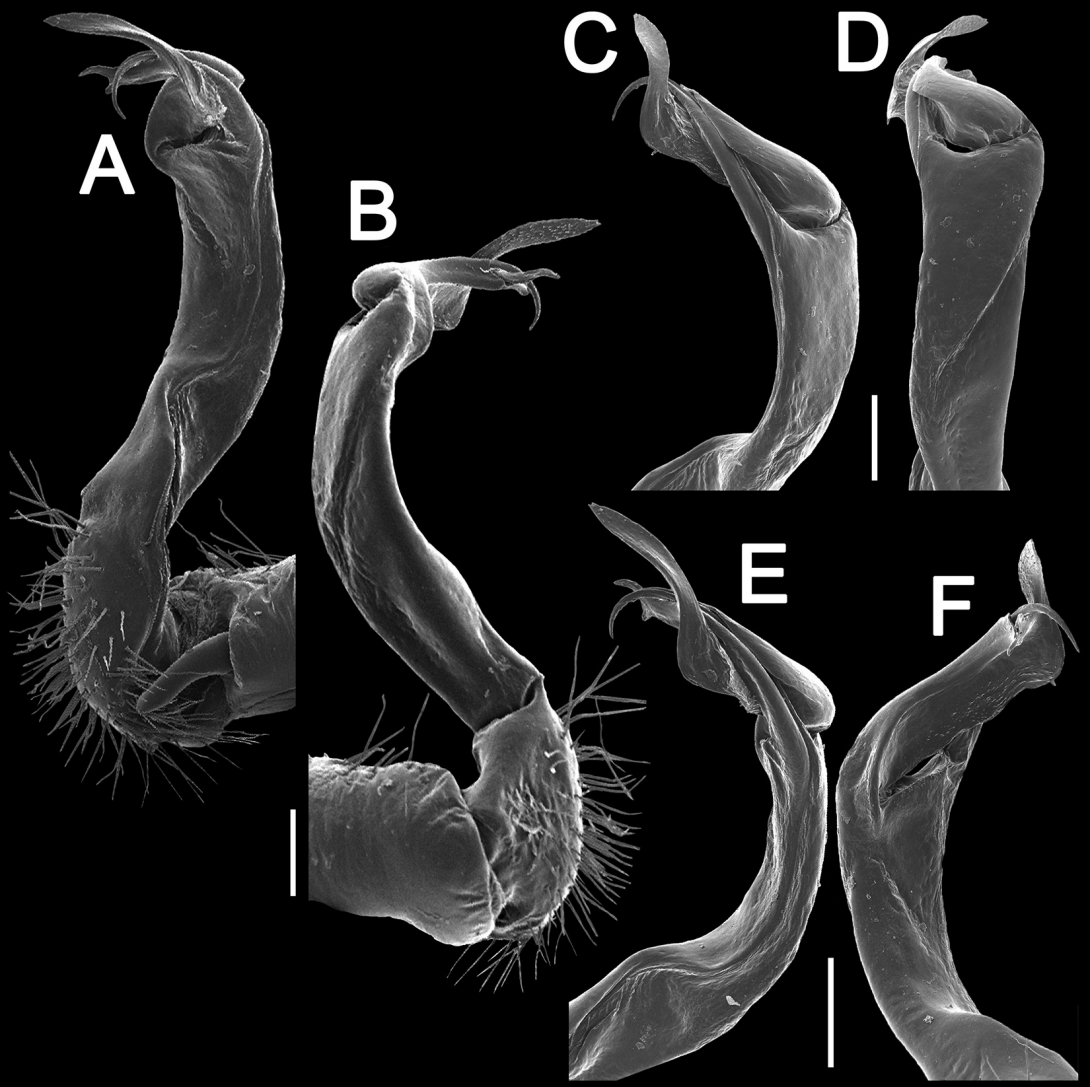
*Antheromorpha rosea* Golovatch, 2013, ♂ from Queen Sirikit Botanic Garden. **A, B** right gonopod, mesal and lateral views, respectively **C–F** distal part of right gonopod, subcaudal, suboral, submesal and sublateral views, respectively. Scale bar: 0.2 mm.

Figs 13 , 14 , 15 , 21

Antheromorpha rosea Golovatch, 2013a: 23 (D).
Antheromorpha rosea – Golovatch 2013b: 308 (D); Nguyen and Sierwald 2013: 1235 (M).

##### Material examined.

5 ♂, 9 ♀ (CUMZ), 1 ♂, 1 ♀ (ZMUM ρ3057), 1 ♂, 1 ♀ (ZMUC), 1 ♂, 1 ♀ (NHMW), Thailand, Chiang Mai Province, Mae Rim District, Queen Sirikit Botanic Garden, 18°53'47"N, 98°51'35"E, ca 640 m a.s.l., 25.09.2014, leg. N. Likhitrakrn. 2 ♂, 30 ♀ (CUMZ), same District, Pong Yang, Ban Muang Kham, 18°53'41"E, 98°49'31.59"E, ca 840 m a.s.l., 20.10.2014, leg. R. Saokord. 1 ♂ (CUMZ), same Province, Hang Dong District, Kaewtachang Waterfall, 18°48'15"E, 98°49'47"E, ca 590 m a.s.l., 24.10.2009, leg. N. Likhitrakarn. 1 ♂ (CUMZ), same Province, Chiang Dao District, Wat Tam Pha Plong, 19°24'13"E, 98°55'16"E, 470 m a.s.l., 28.09.2010, leg. N. Likhitrakarn. 1 ♂ (CUMZ), same Province, Mae Taeng District, Cave Buathong, 19° 4'31.06"N, 99° 5'9.45"E, ca 530 m a.s.l., 22.11.2012, leg. N. Likhitrakarn.

##### Descriptive notes.

Length 33.5–38.0 (♂) or 34.0–44.5 mm (♀), width of midbody pro- and metazonae 2.6–3.5 and 4.4–5.0 mm (♂), 3.2–4.2 and 4.9–5.8 mm (♀), respectively.

Coloration of live animals pinkish (Fig. 13A), with an anterior black band on metaterga and collum; head and antennae blackish, legs dark to light brown; coloration in alcohol, after six months of preservation, faded to light pinkish to pale yellowish (Fig. 13B–H), with a dark brown to blackish brown band on anterior metaterga and collum; head and antennae blackish to light brown, legs light brown to pale yellowish.

Antennae long (Fig. 13A, C), extending behind metaterga 3 when stretched dorsally (♂, ♀). In width, head < segment 3 = 4 < collum < segment 2 < 5–17 (♂, ♀), gently and gradually tapering thereafter (Fig. 13B). Paraterga very strongly developed (Fig. 13B–H), mostly slightly upturned, all lying below dorsum, set at about upper 1/3 of midbody height, caudal corner almost to fully pointed, increasingly acutangular, from narrowly rounded to nearly pointed, especially strongly so in segment 15, thereafter slightly curved mesad (Fig. 13B, D, F). Pleurosternal carinae complete crests with a sharp caudal tooth in segment 2, likewise a sharp caudal tooth in segments 3 and 4, a small, mostly sharp tooth until segment 16 (♂, ♀) (Fig. 13C, E). Sterna delicately and sparsely setose, without modifications, but with a pair of small, rounded, fully separated cones between ♂ coxae 4 (Fig. 13I, J).

##### Remarks.

The available descriptions (Golovatch 2013a, 2013b) of this species were sufficiently detailed to necessitate only a few notes on variation and some new illustrations (Figs 13–15) to show coloration, certain structural details and the gonopod conformation based on new material. This species was described from the ♂ holotype (kept in Senckenberg Museum Frankfurt, Germany) from Gaoligong Shan Moutains, south of Pianma, 25°58'N, 98°40'E, 1600–1700 m a.s.l., Yunnan Province, China (Golovatch 2013a), a little later reported (1 ♂, 1 ♀, deposited in the National Natural History Museum, Sofia, Bulgaria) nearly from the same place (Golovatch 2013b). Even though both these Yunnan localities (Fig. 21) lie far away (ca 730 air-km) from the new Thai records, even despite minor variations, the species identity is beyond doubt.

At least in Thailand, adult *Antheromorpha rosea* have been found to occur every year only for one or two weeks in September or October, disappearing thereafter.

#### Antheromorpha festiva

Taxon classificationAnimaliaPolydesmidaParadoxosomatidae

(Brölemann, 1896)

**Figure 16.**
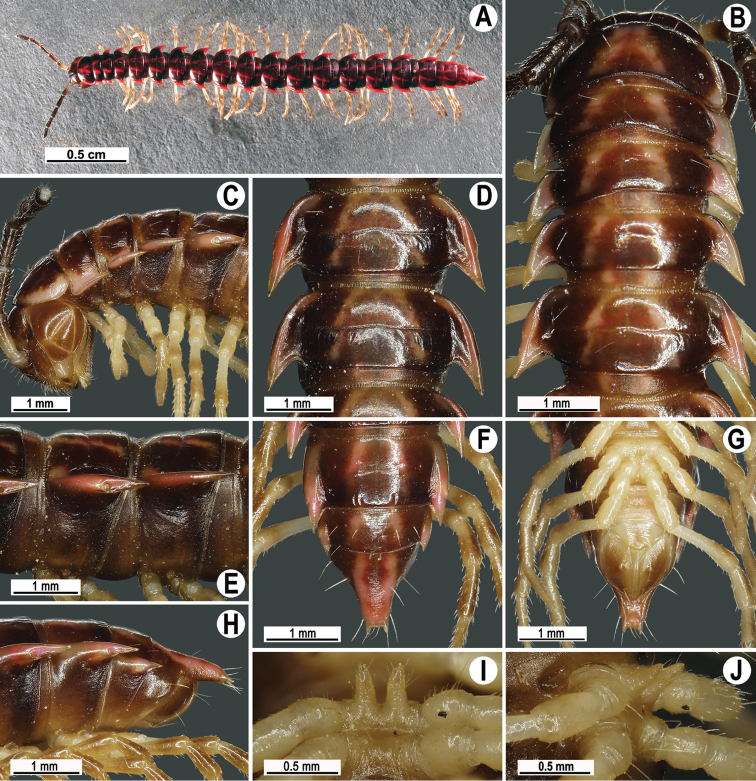
*Antheromorpha festiva* (Brölemann, 1896), ♂ from Sungai Bekok (**A**), ♂ from Kampung S. Ramasamy (**B–J**). **A** habitus, live coloration **B, C** anterior part of body, dorsal and lateral views, respectively **D, E** segments 10 and 11, dorsal and lateral views, respectively **F–H** posterior part of body, lateral, dorsal and ventral views, respectively **I, J** sternal cones between coxae 4, subcaudal and sublateral views, respectively.

**Figure 17.**
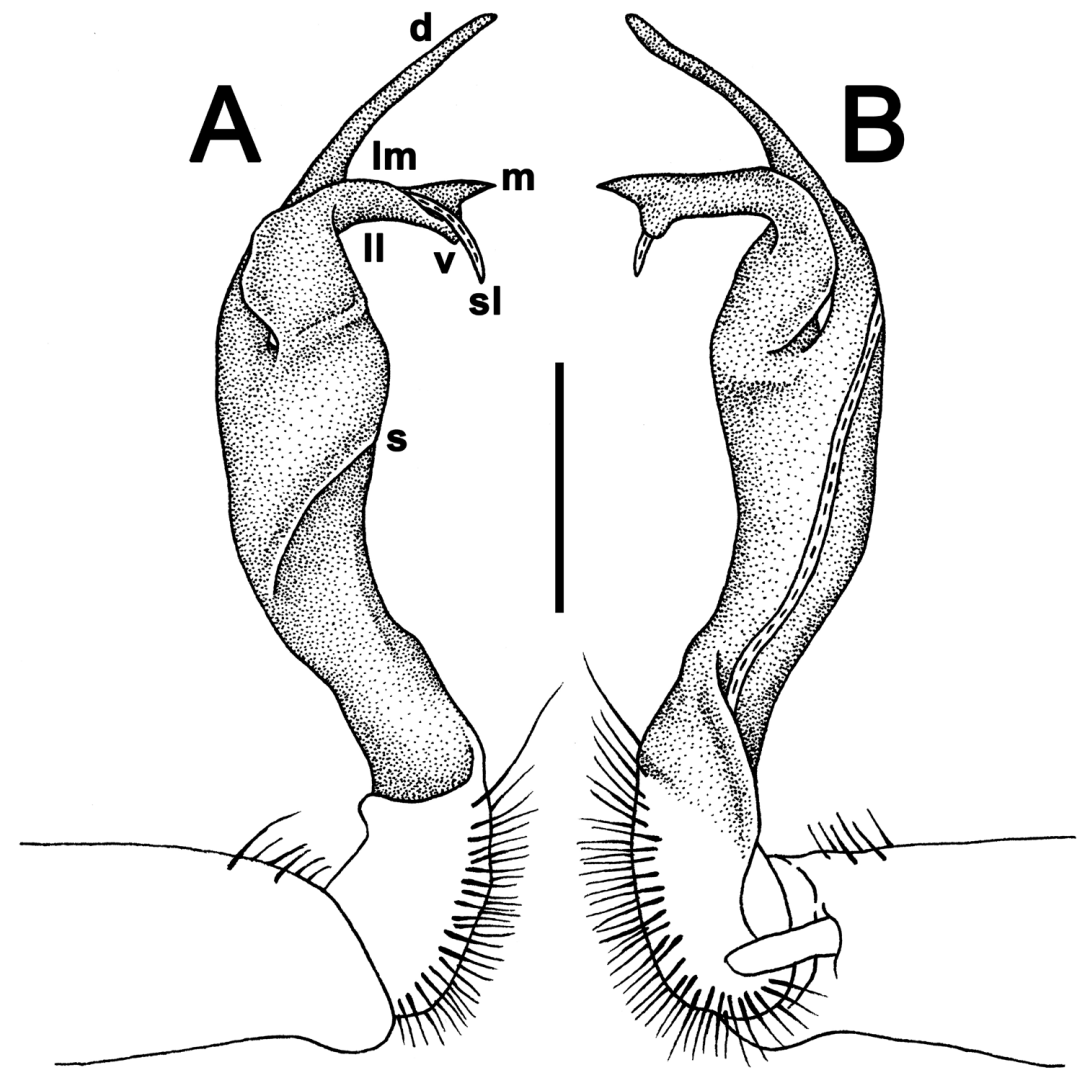
*Antheromorpha festiva* (Brölemann, 1896), ♂ from Kampung S. Ramasamy. **A, B** right gonopod, lateral and mesal views, respectively. Scale bar: 0.4 mm.

**Figure 18.**
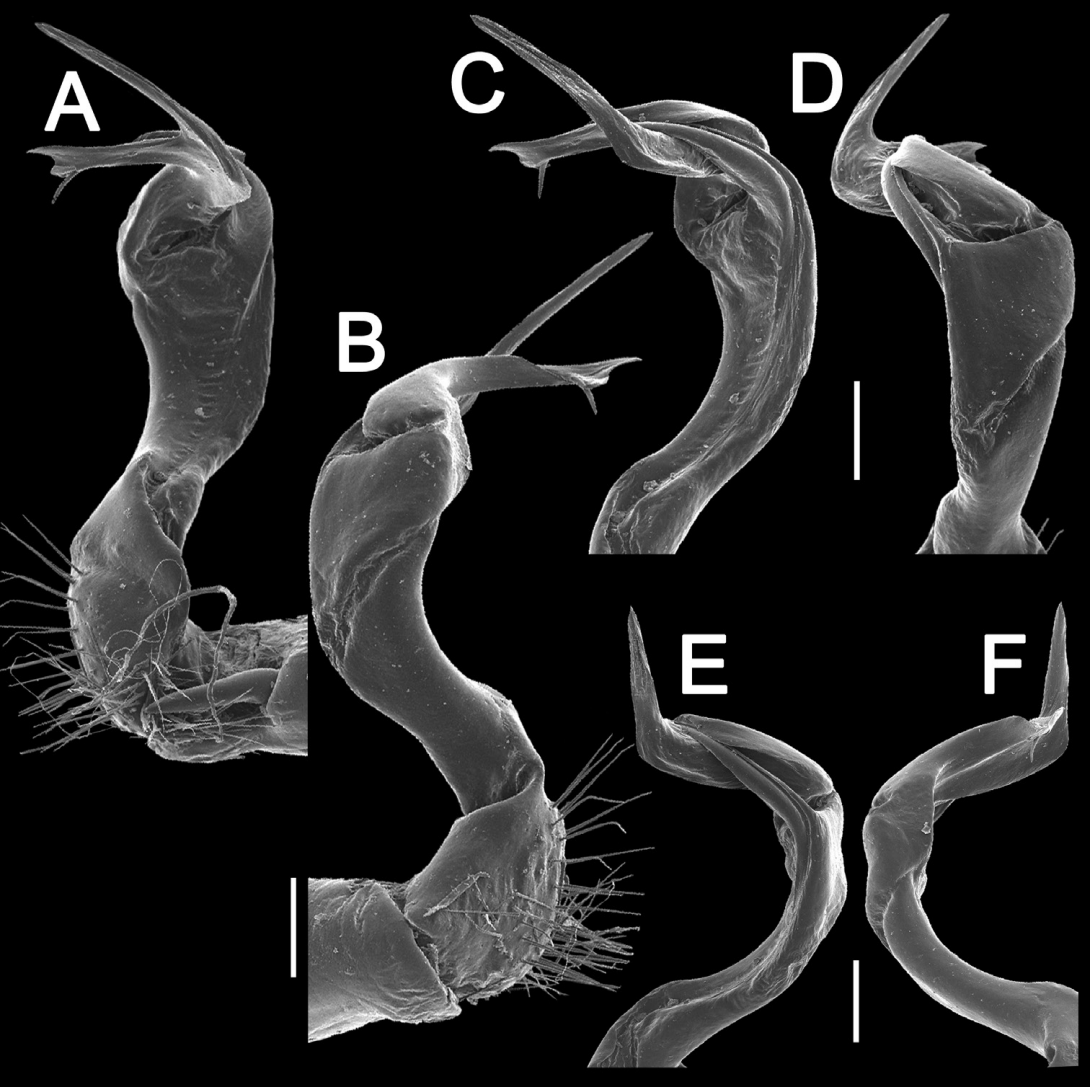
*Antheromorpha festiva* (Brölemann, 1896), ♂ from Kampung S. Ramasamy. **A, B** right gonopod, mesal and lateral views, respectively **C–F** distal part of right gonopod, submesal, sublateral, subcaudal and suboral views, respectively. Scale bar: 0.2 mm.

Figs 16 , 17 , 18 , 21

Orthomorpha festiva Brölemann, 1896: 1 (D).
Orthomorpha festiva – Attems 1898: 339 (M); 1914: 194 (D); 1930: 131 (D); Brölemann 1904: 4 (D, R).
Orthomorpha (Orthomorpha) festiva – Attems 1936: 199 (M); 1937: 60 (D).
“Orthomorpha” festiva – Jeekel 1963: 269 (M).
Antheromorpha festiva – Jeekel 1968: 57 (M); 1980: 85 (M); Golovatch 1983: 181 (M); Enghoff et al. 2004: 37 (M); Enghoff 2005: 95 (R); Nguyen and Sierwald 2013: 1234 (R).

##### Material examined.

3 ♂ (CUMZ), Thailand, Nakhon Si Thammarat Province, Mueang Nakhon Si Thammarat District, Siamthani village, 8°27'53"N, 99°58'10"E, ca 5 m a.s.l., 11.01.2009, leg. N. Likhitrakarn. 1 ♂ (CUMZ), Surat Thani Province, Phanom District, Khao Sok Evergreen House Hotel, 8°54'38"N, 98°31'48"E, leg. C. Sutcharit. 1 ♂ (CUMZ), same Province, Kanchanadit District, Khao Phanom Wang, 9°05'33"N, 99°36'18"E, ca 40 m a.s.l., 15.01.2014, leg. R. Saokord. 12 ♂, 3 ♀ (CUMZ), Satun Province, Mueang Satun District, Wat Kao Noi, 6°45'11"N, 100°01'46"E, ca 40 m a.s.l., 16.01.2014, leg. C. Sutcharit. 7 ♂, 7 ♀ (CUMZ), 2 ♂, 2 ♀ (ZMUM ρ3058), 2 ♂, 2 ♀ (ZMUC), 2 ♂, 2 ♀ (NHMW), same District, Wat Khao Nom Phothiyan, 8°57'22"N, 98°48'20"E, ca 55 m a.s.l., 16.01.2014, leg. R. Saokord and C. Sutcharit. 4 ♂ (CUMZ), Malaysia, Johor, Sungai Bekok, 2°07'11"N, 103°02'25"E, 35 m a.s.l., 21.05.2011, leg. R. Chanabun. 1 ♂ (CUMZ), Perak, Sungai Terong, 4°38'22"N, 100°42'50"E, 30 m a.s.l., 05.06.2014, leg. R. Saokord. 8 ♂, 10 ♀ (CUMZ), 1 ♂, 1 ♀ (ZMUC), 1 ♂, 1 ♀ (NHMW), same state, Kuala Kangsar, Kampung S. Ramasamy, 4°46'55"N, 101°07'14"E, ca 120 m a.s.l., 06.06.2014, leg. R. Saokord.

##### Redescription.

Length 23.0–29.5 (♂) or 26.0–34.5 mm (♀), width of midbody pro- and metazonae 1.8–2.5 and 2.9–3.7 mm (♂), 2.7–3.1 and 3.6–4.4 mm (♀), respectively (vs length 28–30 mm, as given in the available descriptions (Brölemann 1896; Attems 1937).

Coloration of live animals dark red to red-brownish, with contrasting light red to pale pinkish paraterga and epiproct; a complete inverted V-shaped line on collum, a pair of parallel oblique bands on metazonae and a pair of parallel bands on prozonae of following segments; legs and venter brownish to pale brown; coloration of alcohol material after one year of preservation faded to castaneous or pale brown; paraterga, epiproct and parallel bands faded to pale pinkish or pale yellow, legs and venter paler brown to yellowish (Fig. 16B–J).

Clypeolabral region sparsely setose, epicranial suture distinct. Antennae short (Fig. 16A), reaching anterior edge of body segment 3 (♂) or 2 (♀) when stretched dorsally (antennomere 6 broadest). In width, head < collum < segment 2 < 3 < 4 < 5–16, gently and gradually tapering thereafter. Collum with three transverse rows of setae: 3+3 in anterior, 1+1 in intermediate and 3+3 in posterior row, the latter mostly traceable as insertion points; caudal corner broadly rounded, slightly bordered and declined ventrally, not extending behind tergal margin (Fig. 16B, C).

Tegument smooth and shining, prozonae very finely shagreened, metazonae smooth and delicately rugulose; surface below paraterga finely microgranulate. Postcollum metaterga with two transverse rows of setae, these being always abraded and traceable as insertion points: 2+2 in anterior (pre-sulcus) row, 3+3 in posterior (post-sulcus) one. Tergal setae simple, slender, about 1/3 of metatergal length. Axial line visible both on pro- and metazonae, starting with collum. Paraterga very strongly developed (Fig. 16A–H), subhorizontal, all lying below dorsum, set at about upper 1/3 of midbody height, anterior edge of paraterga broadly rounded, bordered and fused to callus; lateral edge of paraterga 2 with three small incisions, but on following segments smooth with only insertion points of setae (at fore 1/4), mostly abraded; caudal corner almost completely to fully pointed, always extending behind rear tergal margin, bent posteriad on segments 17 and 18; posterior edge evidently concave (Fig. 16B, D, F). Calluses delimited by a sulcus both dorsally and ventrally. Ozopores evident, lateral, lying in an ovoid groove at about 1/2 of metatergite’s length. Transverse sulcus usually distinct (Fig. 16B, D–F), complete on metaterga 5–17, incomplete on segments 4 and 18, narrow, wave-shaped, not reaching bases of paraterga, faintly beaded at bottom. Stricture between pro- and metazonae wide, deep, beaded at bottom down to base of paraterga (Fig. 16B–F). Pleurosternal carinae complete crests only on segments 2–4, each with an evident sharp denticle caudally on segments 5–8 (♂, ♀), thereafter increasingly reduced until segment 13 (♂) or 10 (♀). Epiproct (Fig. 16F–H) conical, flattened dorsoventrally, with two evident apical papillae, tip subtruncate; pre-apical papillae small, but visible. Hypoproct (Fig. 16G) roundly subtriangular, setiferous knobs at caudal edge well-separated.

Sterna sparsely setose, without modifications; a high paramedian pair of evident, high, nearly pointed, fully separated, setose cones between ♂ coxae 4 (Fig. 16I, J). Legs moderately long and slender, midbody ones ca 1.2–1.4 (♂) or ca 1.0–1.2 times (♀) as long as body height, prefemora without modifications, ♂ tarsal brushes present until segment 16.

Gonopods (Figs 17, 18) with femorite relatively short and rather stout, evidently curved and enlarged distad, postfemoral portion demarcated by an oblique lateral sulcus; tip of solenophore (**sph**) very deeply bifid, with a long, slender, nearly pointed process **d**; process **m** with an acute terminal lobule, longer than a small and terminally rounded process **v**.

##### Remarks.

The new specimens fully agree with the most detailed and beautifully illustrated redescription of the species as given by Brölemann (1904), whereas the original description (Brölemann 1896) was indeed so concise and contained no type locality other than “Indo-Chine” that Attems (1937), obviously being unaware of Brölemann’s 1904 paper, reiterated only the very short diagnosis of *Orthomorpha festiva* contained in Brölemann (1896). According to Brölemann (1904), however, this species (1 ♂ and 1 ♀ syntypes, now in the Paris Museum) actually derived from “Siam”. Enghoff et al. (2004), likewise unaware of Brölemann’s (1904) detailed redescription, erroneously listed *Antheromorpha festiva* as coming from “southern Vietnam”, but very soon after that the mistake was corrected for “Siam” (Enghoff 2005).

The above samples thus derive from the first specified localities in Thailand. Moreover, *Antheromorpha festiva* appears to be not only new to the fauna of Malaysia, but it also seems to be quite widespread across the southern half of Malay Peninsula both within lowland southern Thailand and Western Malaysia, being confined there to elevations not exceeding 60 m a.s.l. (Fig. 21).

#### Antheromorpha harpaga

Taxon classificationAnimaliaPolydesmidaParadoxosomatidae

(Attems, 1937)

Figs 19 , 20 , 21

Orthomorpha harpaga Attems, 1937: 77 (D).
Orthomorpha harpaga – Attems 1938: 211 (D).
“Orthomorpha” harpaga – Jeekel 1963: 269 (M).
Antheromorpha harpaga – Jeekel 1968: 57 (M).
Antheromorpha harpaga – Jeekel 1980: 85 (M); Golovatch 1983: 181 (M); Enghoff et al. 2004: 37 (M); Nguyen and Sierwald 2013: 1234 (M).

##### Lectotype

♂ of *Orthomorpha harpaga* (NHMW-3495), Vietnam, Khánh Hòa Province, 15 km southwest of Ngatrang, Souidau (= Cam Lam-Suoi Cat 1), 06.1933, leg. C. Dawydoff.

##### Paralectotype.

1 ♂ (NHMW-3495), same locality, together with lectotype.

The lectotype is designated here to ensure that the name-bearing specimem is a complete ♂.

##### Redescription.

**Figure 19.**
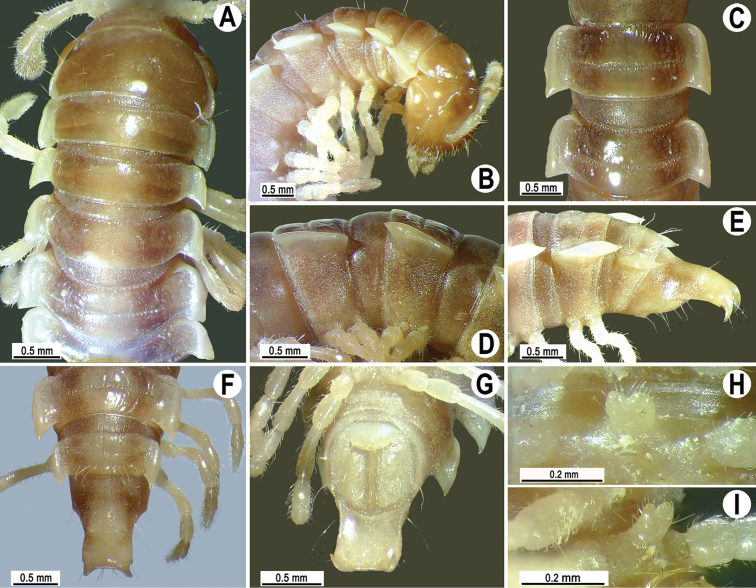
*Antheromorpha harpaga* (Attems, 1937), ♂ lectotype. **A, B** anterior part of body, dorsal and lateral views, respectively **C, D** segments 10 and 11, dorsal and lateral views, respectively **E–G** posterior part of body, lateral, dorsal and ventral views, respectively **H, I** sternal cones between coxae 4, subcaudal and sublateral views, respectively.

Length 19–21 mm (♂), width of midbody pro- and metazonae 1.8–1.9 and 2.3–2.6 mm, respectively (vs 1.8 and 2–2.5 mm in width of pro- and metazonae, respectively, as given in the available descriptions (Attems 1937, 1938)). Coloration in alcohol, after long-term preservation, uniformly brown with a pale yellowish median stripe (Fig. 19A–F), paraterga and epiproct pale whitish yellow or pale brown; antennae, legs and sterna whitish to pale brown.

Clypeolabral region sparsely setose, epicranial suture distinct. Antennae short (Fig. 19A), clavate (antennomere 6 broadest), reaching anterior edge of body segment 3 when stretched dorsally. In width, head < collum < segment 3 = 4 < segment 2 < 5–17, gently and gradually tapering thereafter. Collum with three transverse rows of setae: 4+4 in anterior, 3+3 in intermediate and 3+3 barely traceable insertion points in posterior row; caudal corner broadly rounded, slightly bordered and declined ventrally, not extending behind tergal margin (Fig. 19A, B).

Tegument smooth and finely shargreened, metaterga smooth and delicately rugulose; surface below paraterga finely microgranulate. Postcollum metaterga with two transverse rows of setae traceable at least as insertion points when setae broken off: 2+2 in anterior (pre-sulcus), 3+3 in posterior (post-sulcus) row. Tergal setae simple, slender, about 1/3 of metatergal length. Axial line barely visible, starting with collum. Paraterga very strongly developed (Fig. 19A–G), slightly upturned, all lying below dorsum, set at about upper 1/3 of midbody height, anterior edge of paraterga broadly rounded, bordered and fused to callus; lateral edge of paraterga 2 with three small incisions, with two small incisions in anterior half on poreless segments, with only one incision near front 1/3 on pore-bearing ones; caudal corner of paraterga narrowly rounded, increasingly well pointed on paraterga 16–19; paraterga bent posteriad, extending behind tergal margin; posterior edge oblique. Calluses delimited by a sulcus only dorsally. Ozopores evident, lateral, lying in an ovoid groove at about 1/4 of metatergite’s length in front of caudal corner. Transverse sulcus usually distinct (Fig. 19A, C, F), complete on metaterga 5–18, incomplete on segment 19, wide, line-shaped, reaching bases of paraterga, evidently ribbed at bottom. Stricture between pro- and metazonae wide, shallow, clearly ribbed at bottom down to base of paraterga (Fig. 19A–F). Pleurosternal carinae complete crests with a sharp caudal tooth on segments 2–4, bulged anteriorly and with a sharp caudal tooth on segments 5–7, thereafter only a small, sharp, caudal tooth on segments 8–11 (Fig. 19B, D, E). Epiproct (Fig. 19E–G) large, subrectangular, flattened dorsoventrally, with two apical papillae remarkably curved caudoventrally, claw-shaped; tip subtruncate; pre-apical papillae small, but visible, lying rather close to tip. Hypoproct semi-circular, setiferous knobs at caudal edge well-separated.

Sterna sparsely setose, without modifications; a high, subcordiform, sternal lobe between ♂ coxae 4 (Fig. 19H, I). Legs moderately long and slender, midbody ones ca 1.2–1.4 times as long as body height, prefemora without modifications, ♂ tarsal brushes present until legs of segment 18.

**Figure 20.**
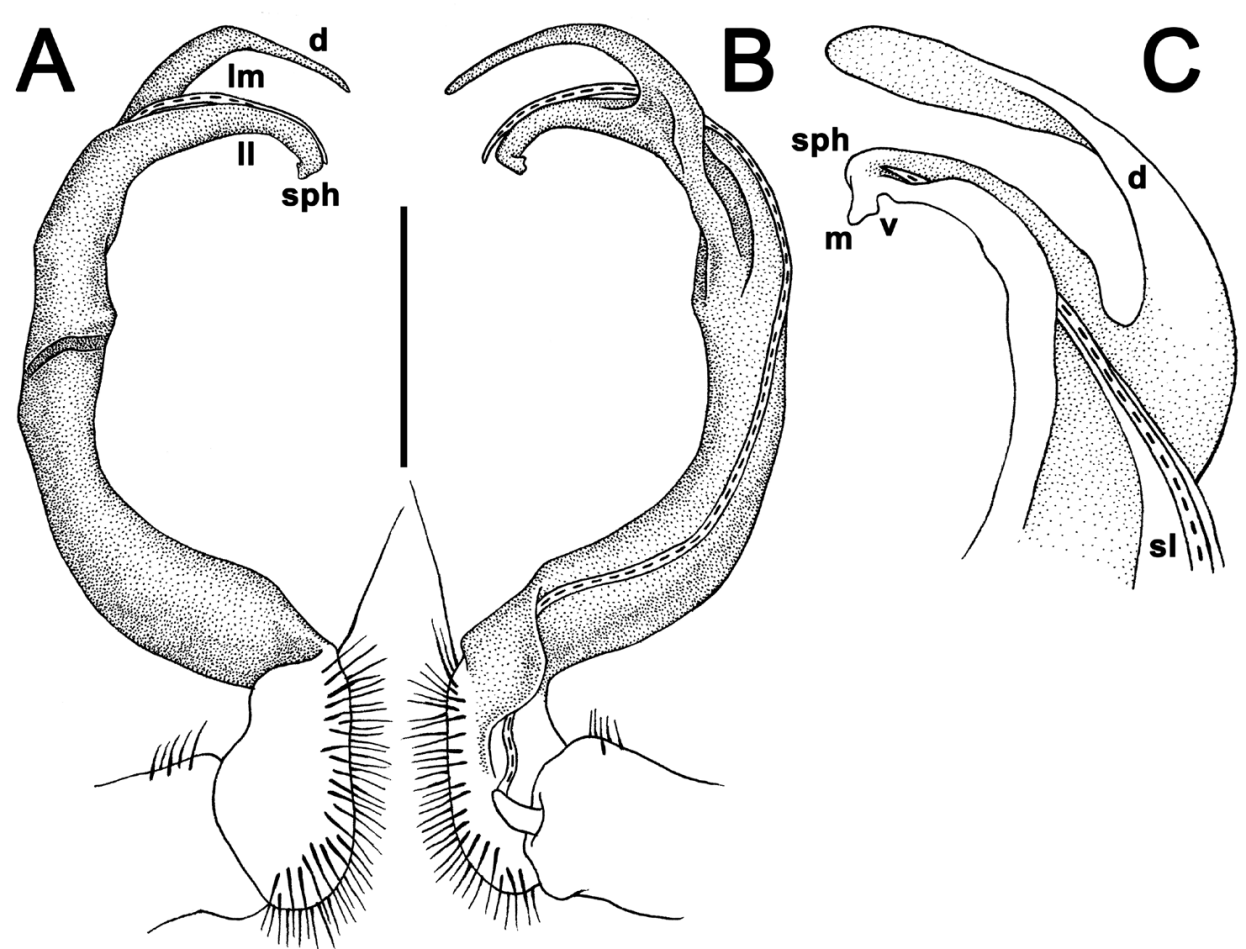
*Antheromorpha harpaga* (Attems, 1937), ♂ lectotype. **A, B** right gonopod, lateral and mesal views, respectively **C** left gonopod, lateral view. Scale bar: **A, B** 0.4 mm **C** drawn not to scale.

Gonopods (Fig. 20) long and slender. Prefemoral part about 3 times shorter than femorite (= strongly setose) part. Femorite slender, evidently curved, postfemoral part demarcated by an oblique lateral sulcus; tip of solenophore (**sph**) clearly deeply bifid, with a very long, slender, pointed process (**d**); processes **m** and **v** very small tubercles.

**Figure 21.**
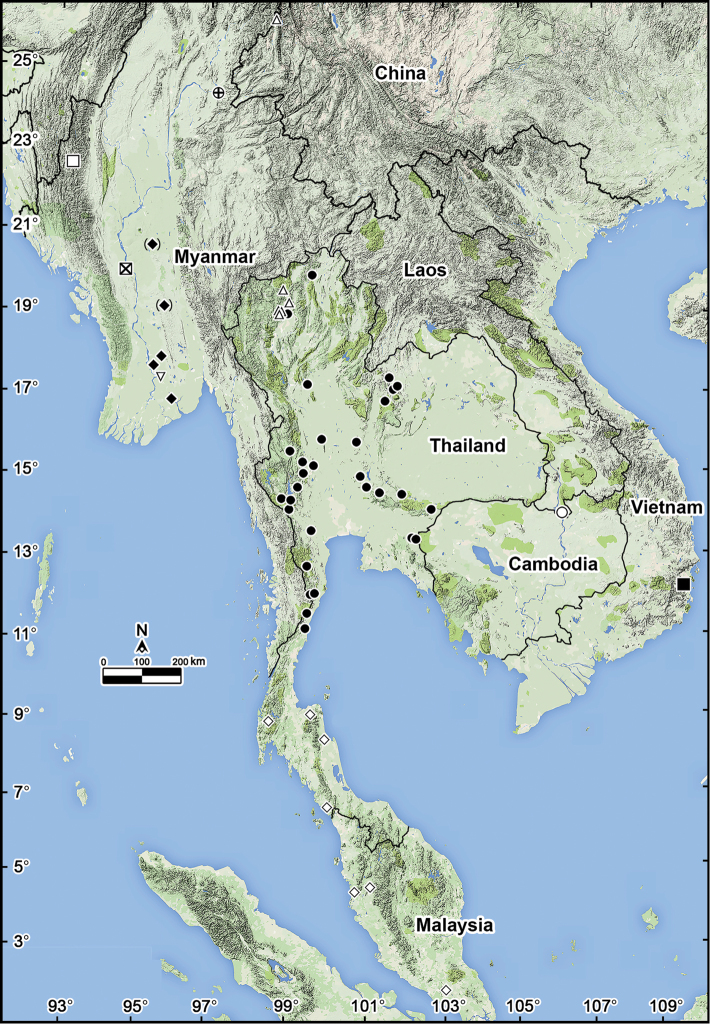
Distribution of *Antheromorpha* species (11 species). **Open triangle** *Antheromorpha rosea* Golovatch, 2013 **Crossed circle** *Antheromorpha bistriata* (Pocock, 1895) **Open square** *Antheromorpha comotti* (Pocock, 1895) and *Antheromorpha mediovirgata* (Carl, 1941) **Filled diamond** *Antheromorpha miranda* (Pocock, 1895) **Crossed square** *Antheromorpha comotti* (Pocock, 1895), *Antheromorpha miranda* (Pocock, 1895) and *Antheromorpha minlana* (Pocock, 1895) **Inverted open triangle** *Antheromorpha pardalis* (Pocock, 1895) **Filled Circle** *Antheromorpha uncinata* (Attems, 1931) **Open Circle** *Antheromorpha paviei* (Brölemann, 1896) **Filled square** *Antheromorpha harpaga* (Attems, 1937) **Open diamond** *Antheromorpha festiva* (Brölemann, 1896).

##### Remarks.

This is the only species in this genus that has been reported from Vietnam (Attems 1937). It differs from congeners in the gonopod solenophore being deeply bifid and showing a long and slender process **d** and a bidentate tip (Fig. 20C).

#### Key to the known species of *Antheromorpha*, chiefly based on ♂.

**Table:** 

1	Colour pattern of metaterga: yellowish paramedian spots in front of transverse sulcus, the latter visible starting with segment 2	***Antheromorpha pardalis***
–	Colour pattern of metaterga otherwise. Transverse sulcus present starting with segment 4 or 5	**2**
2	Colour pattern of metaterga: yellowish paramedian stripes	**3**
–	Colour pattern of metaterga otherwise	**8**
3	Gonopod femorite relatively short (Figs 2A, 3A, 17, 18A, B)	**4**
–	Gonopod femorite longer (Figs 1D, E, 11A, B, 14, 15A, B, 20A, B)	**6**
4	Metazonae ca 2.0 mm wide. Pleurosternal carinae poorly developed, in ♂ slightly projecting caudad behind rear tergal margin only until segment 5	***Antheromorpha mediovirgata***
–	Metazonae ≥ 2.9 mm wide. Pleurosternal carinae well-developed, in ♂ slightly projecting caudad behind rear tergal margin at least until segment 10	**5**
5	Sternal lamina between ♂ coxae 4 with a paramedian pair of evident, high, nearly pointed, fully separated, setose cones (Fig. 16I, J). Gonopod process **d** very long (Figs 17A, B, 18A, B). Southern Thailand and Western Malaysia (Fig. 21)	***Antheromorpha festiva***
–	Sternal lamina between ♂ coxae 4 with only single small cone. Gonopod process **d** shorter (Fig. 2A). Northern Myanmar (Fig. 21)	***Antheromorpha bistriata***
6	Sternal lamina between ♂ coxae 4 a large, cordiform, ventrally evidently concave lobe (Fig. 2G). Gonopod solenophore longer and rather straight (Fig. 2H)	***Antheromorpha comotti***
–	Sternal lamina between ♂ coxae 4 a simple, rounded, conical knob. Gonopod solenophore evidently curved (Figs 1D, E, 3C)	**7**
7	Metazonae ca 2.6 mm (♂) or ca 3.0 mm wide (♀). Gonopod femorite rather straight, process **d** longer than solenophore (**sph**) (Fig. 3C)	***Antheromorpha minlana***
–	Metazonae 3.2–3.7 mm (♂) or 3.6–4.6 mm wide (♀). Gonopod femorite strongly curved caudad, process **d** shorter than solenophore (**sph**) (Fig. 1D, E)	***Antheromorpha miranda***
8	Sternum between ♂ coxae 4 with a single lamina or cone (Figs 8H, I, 9H, I, 19H, I)	**9**
–	Sternum between ♂ coxae 4 with a pair of separated cones (Figs 4I, J, 13I, J)	**10**
9	Colour pattern: a light axial stripe flanked on each side by a dark stripe on collum to epiproct (Figs 7, 8A, C, 9A, C, J–L). Epiproct simple, not especially large, with two small, but evident apical papillae (Figs 8E–G, 9E–G). Tip of gonopod split rather deeply, but process **d** shorter (Figs 10–12). Thailand (Fig. 21)	***Antheromorpha uncinata***
–	Colour pattern indistinct, with a pale yellowish median stripe against a uniformly brown background (Fig. 19A–F). Epiproct particularly large, with two apical papillae curved remarkably ventrad, claw-shaped (Fig. 19E–G). Tip of gonopod split deeper, process **d** very long (Fig. 20). Southern Vietnam (Fig. 21)	***Antheromorpha harpaga***
10	Colour pattern: paraterga and epiproct contrasting dark yellow on a blackish body (Fig. 4A–H). Caudal corner of paraterga on anterior body part almost or fully pointed (Fig. 4A–H). ♂ tarsal brushes present until segment 8. Southern Laos (Fig. 21)	***Antheromorpha paviei***
–	Colour pattern: a dark band present only on posterior halves of proterga and posterior halves of metaterga (Fig. 13A–H). Caudal corner of paraterga on anterior body part narrowly rounded (Fig. 13A–H). ♂ tarsal brushes present until segment 17. Southern China and northern Thailand (Fig. 21)	***Antheromorpha rosea***

## Conclusions

As a result of our review, the genus *Antheromorpha* now comprises 11 species ranging from southern China, through Myanmar, Thailand, Laos and Vietnam, to Western Malaysia (Fig. 21). Alloparty seems to be prevailing if not complete. Even though Myanmar alone supports as many as six species of *Antheromorpha*, with *Antheromorpha miranda*, *Antheromorpha comotti* and *Antheromorpha minlana* co-occurring at Minhla, *Antheromorpha comotti* and *Antheromorpha mediovirgata* in the Chin Hills and *Antheromorpha miranda* and *Antheromorpha pardalis* at Palon/Pegu, their strict sympatry remains questionable as the records from Myanmar are very old and thus somewhat uncertain. The much better explored Thailand harbours three apparently strictly allopatric species. Even in Chiang Mai Province, where *Antheromorpha uncinata* and *Antheromorpha rosea* co-occur, allopatry looks complete, without any mixed populations observed.

The pair *Antheromorpha uncinata* and *Antheromorpha festiva* shows a remarkable geographical gap in southern Thailand, more specifically, in the northern half of the Malay Peninsula (Fig. 21). This gap strongly reminds of that observed between *Orthomorpha lauta* Golovatch, 1998 and *Orthomorpha insularis* Pocock, 1895 (Likhitrakarn et al. 2011).

There can be no doubt that further collecting efforts, especially in still very poorly explored regions such as Laos, China, Malaysia and Vietnam, will reveal more *Antheromorpha* species, as well as further records of the known congeners. Cambodia remains an especially poorly prospected country in Indochina whence no *Antheromorpha* has been documented yet (Likhitrakarn et al. 2015).

## Acknowledgements

This project was partly funded through grants received from the Office of the Royal Development Projects Board, while most of the financial support was obtained from The Thailand Research Fund, The TRF Senior Research Scholar RTA 5880002 (2015–2018) to SP. We thank the members of the Animal Systematics Research Unit for their invaluable assistance in the field. Both Henrik Enghoff (ZMUC) and Thomas Wesener (Zoologisches Forschungsmuseum Alexander Koenig, Bonn, Germany) kindly provided thorough reviews of an advanced draft.

## Supplementary Material

XML Treatment for Antheromorpha

XML Treatment for Antheromorpha miranda

XML Treatment for Antheromorpha bistriata

XML Treatment for Antheromorpha comotti

XML Treatment for Antheromorpha mediovirgata

XML Treatment for Antheromorpha minlana

XML Treatment for Antheromorpha pardalis

XML Treatment for Antheromorpha paviei

XML Treatment for Antheromorpha uncinata

XML Treatment for Antheromorpha rosea

XML Treatment for Antheromorpha festiva

XML Treatment for Antheromorpha harpaga
